# Kinetics and pathways of sub‐lithic microbial community (hypolithon) development

**DOI:** 10.1111/1758-2229.13290

**Published:** 2024-06-23

**Authors:** Jason Bosch, Pedro H. Lebre, Eugene Marais, Gillian Maggs‐Kölling, Don A. Cowan

**Affiliations:** ^1^ Centre for Microbial Ecology and Genomics, Department of Biochemistry, Genetics and Microbiology University of Pretoria Pretoria South Africa; ^2^ Institute of Microbiology of the Czech Academy of Sciences Czech Academy of Sciences Praha Czech Republic; ^3^ Gobabeb–Namib Research Institute Walvis Bay Namibia

## Abstract

Type I hypolithons are microbial communities dominated by Cyanobacteria. They adhere to the underside of semi‐translucent rocks in desert pavements, providing them with a refuge from the harsh abiotic stresses found on the desert soil surface. Despite their crucial role in soil nutrient cycling, our understanding of their growth rates and community development pathways remains limited. This study aimed to quantify the dynamics of hypolithon formation in the pavements of the Namib Desert. We established replicate arrays of sterile rock tiles with varying light transmission in two areas of the Namib Desert, each with different annual precipitation regimes. These were sampled annually over 7 years, and the samples were analysed using eDNA extraction and 16S rRNA gene amplicon sequencing. Our findings revealed that in the zone with higher precipitation, hypolithon formation became evident in semi‐translucent rocks 3 years after the arrays were set up. This coincided with a Cyanobacterial ‘bloom’ in the adherent microbial community in the third year. In contrast, no visible hypolithon formation was observed at the array set up in the hyper‐arid zone. This study provides the first quantitative evidence of the kinetics of hypolithon development in hot desert environments, suggesting that development rates are strongly influenced by precipitation regimes.

## Graphical abstract

Analysis of the microbial composition of desert sub‐lithic hypolithon communities growing under semi‐translucent rocks over a period of 7 years has provided the first quantitative evidence of a timeline for early hypolithon development. Data from this study has led us to propose a hypolithon development involving initial recruitment of specific soil Cyanobacteria to the illuminated soil surface immediately underneath a translucent rock followed by a transition to an adherent state triggered by wet‐dry cycles, and further maturation to hypolithon biofilm.

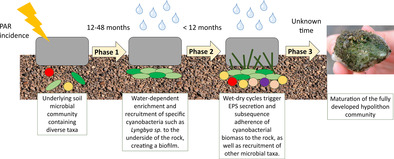

## INTRODUCTION

Deserts are extreme but widespread environments (Mirzabaev et al., 2019), where life must subsist under intense abiotic stress, including water deficit and extreme temperature variations (Lebre et al., 2017; Ward, 2010). As a consequence, deserts typically harbour a limited range of specialized animals and plants, while ecosystem services are largely driven by microbial communities living in the soil (Belnap, 2006; Makhalanyane et al., 2015; Pointing & Belnap, 2012) or in cryptic edaphic niches (Cowan et al., 2020).

Hypolithons inhabit the cryptic niche of the ventral surface of semi‐translucent rocks, where they are partially protected from the abiotic stresses of the desert soil surface (Chan et al., 2012; Lebre et al., 2021). The overlying semi‐translucent rock, most commonly quartz, filters short wavelength solar radiation, buffers temperature extremes and maintains a higher relative humidity than the surface atmosphere (Azúa‐Bustos et al., 2011; Cockell & Stokes, 2006; Ekwealor & Fisher, 2020). In deserts where quartz rocks are common elements of mineral pavements, hypolithons have been shown to be a ubiquitous feature of the landscape (Azúa‐Bustos et al., 2011; Warren‐Rhodes et al., 2006). In the Namib Desert, colonization rates of quartz rocks in the desert pavement have been estimated at approximately 98% (Warren‐Rhodes et al., 2013). This colonization occurs over a natural longitudinal water gradient across the desert that is characterized by two main sources of water. In the coastal regions, regular fog events provide up to 183 mm of moisture (per annum) as the only regular water input, up to 60 km eastwards (Eckardt et al., 2013; Lancaster et al., 1984). Beyond this point, rainfall frequency and intensity increase towards the eastern margins of the desert (from 15 to 185 mm per annum west‐to‐east) (Eckardt et al., 2013; Lancaster et al., 1984). This natural xeric gradient has led to high level of species endemism, including the coast lichen communities (Hinchliffe et al., 2017), and fog‐harvesting beetles (Hamilton & Seely, 1976).

Hypolithic communities can make a large contribution to nutrient cycling of the surrounding soils (Cockell & Stokes, 2004, Ramond et al., 2018: Cowan et al., 2011). Type I hypolithons, dominated by Cyanobacteria, are the most documented hypolith structures around the globe (Caruso et al., 2011; Lacap‐Bugler et al., 2017). Moss‐dominated hypolithons (Cowan et al., 2010) are uncommon, but have been hypothesised to represent successional stages of hypolith development (Makhalanyane et al., 2013).

Despite their importance to desert ecosystems, very little is known about hypolithon growth rates or hypolithic community development pathways, as well as how these are impacted by water availability. Attempts have been made to understand the processes affecting community assembly at various scales (Caruso et al., 2011; Lebre et al., 2020; Warren‐Rhodes et al., 2013), but these have not taken into account the timeline of development. In order to quantitate and understand the dynamics of hypolithon formation as well as account for the impact of water availability, we established replicate arrays of sterile, semi‐translucent, and opaque rocks in two Namib Desert pavement sites across the west–east precipitation gradient. Annual sampling over a period of 7 years, coupled with eDNA extractions and 16S rRNA gene amplicon sequencing, has allowed us to assess the dynamics and kinetics of hypolithon formation.

## EXPERIMENTAL PROCEDURES

### Hypolith array and sample collection

**FIGURE 1:**
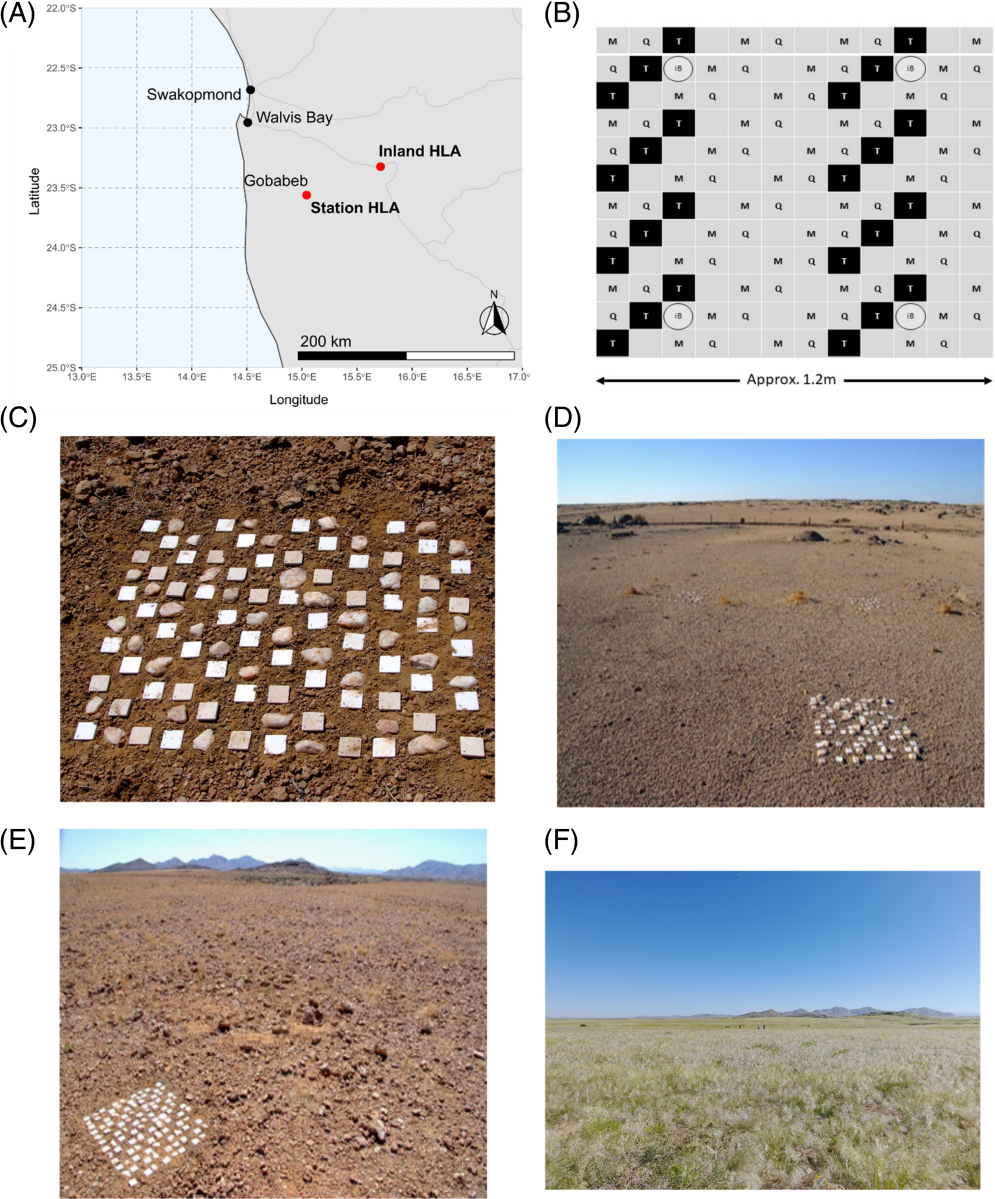
(A) Map of Namibia showing the locations of the hypolith arrays, represented by the red points labelled as station array and Inland array. (B) Array conformation scheme used for each array in this study. Tiles are labelled using the following nomenclature: Tile: M, marble tile; Q, quartz rock; T, travertine tile; iB, iButton placement (0.5 cm depth). (C) A hypolith array as constructed in 2015. (D–E) Landscape view of the station (D) and the inland (E) arrays. (F) Site of the inland array after a period of abnormally high rainfall.

Two 1.2 m × 1.2 m rock arrays, consisting of 104 rocks of three different types, were established in the Namib Desert in 2015. The inland array (inland HLA) was established in the eastern rainfall zone (S 23.32325, E 15.71369) and station array (station HLA) was positioned in the hyper‐arid central zone, near the Gobabeb‐Namib Research Institute (S 23.56056, E 15.04215) (Figure 1A).

Meteorologic data recorded by the SASSCAL WeatherNet stations (http://www.sasscalobservationnet.org/index.php) closest to the array sites (Gobabed station: S 23.560437, E 15.040998 at the location of the station array; Ganab station: S 23.12182, E 15.53844, 28 km from the inland array) showed that the inland array had a mean annual precipitation of 5.4 mm between 2015 and 2022, with 21 months exhibiting mean monthly precipitation >10 mm. By contrast, the station array had a mean annual precipitation of 1.2 mm between 2015 and 2022, with only 4 months recording mean monthly precipitations >10 mm (Figure S1).

Three different rock types, quartz, marble, and travertine, were positioned at each location following a predetermined arrangement (Figure 1B, C). Natural quartz pebbles were collected from the central Namib Desert (near Mirabeb: S 23.45351, E 15.35382) and cleaned by removal of soil and hypolithic biomass. All natural local quartz rocks used in the array assemblies were sized between 5–8 cm (length and width) and 2–4 cm depth. Tiles of marble and travertine, cut to 6 cm × 6 cm × 1 cm, were a gift from M. Wing (California, USA). Travertine tiles were used as a ‘dark rock’ control, due to being 100% opaque. The light (PAR) transmission of all tiles and natural quartz pebbles was measured using a LiCor LI 190R quantum sensor. Light transmission readings were taken for each item in four different orientations (N, S, E, W) and compared to unrestricted solar PAR at the same time.

All rocks and tiles were oven‐sterilized at 180°C for 2 h and transported under aseptic conditions prior to assembly of the arrays. All tiles and rocks were embedded in the soil to a depth of 0.5–1 cm. The landscape surrounding the arrays is characterized as gravel pavement with scarce to no vegetation (Figure 1D, E), which reflects the precipitation regimes of the chosen locations. The soil substrate at both sites consisted of unaltered entisols typical of many desert surfaces (Eckardt et al., 2022), which consists of unaltered sediments accumulating from in situ weathering of older sediments or underlying geological formations and aeolian dust deposition. Although the locations were selected away from any rock outcrops where entisols covered the underlying geology, the local geology at both sites consists of granite intermixed with steep sloping metamorphic schists and thin quartz veins. There was no visual indication of biocrusts at these locations, such as crustose lichens, mosses or visible thallus structures, while epilithic lichen occurrences in the areas are confined to the uppermost edges of protrusions at rock outcrops and does not form part of the pedoderm (sensu Eckardt et al., 2022). Perennial vegetation cover is markedly absent at the selected locations, with no shrubs within 10 meters of the arrays. There was no evidence of perrenial grass near the Station array, while sparse stubble of dead tussocks, some within 1 meter of the inland array, indicate the occasional occurrence of perennial grass at more inland locations. As indicated in Figure 1F, in March 2022, eastern regions of the Namib Desert were subject to abnormally high levels of precipitation (21.3 mm mean monthly precipitation), resulting in a drastic increase in transient vegetation density at the inland array site.

The hypolith arrays and nearby open soils (control soils) were sampled annually for a period of 7 years, from 2015 to 2022, with no sampling performed in 2020 because of travel restrictions due to the COVID‐19 pandemic. In 2015, at the time of array assembly, only control soils samples were recovered (in triplicate). From 2016 onward, in April of each year, the surrounding soil and three rock types (quartz, marble, and travertine) were sampled in triplicate. The sampling procedure involved recovering the rock (with any adherent material) and 0.5 cm of soil from below the embedded rock, into a sterile Whirl‐Pak® bag. Sampling was performed randomly across the array, to avoid sampling bias. In 2022, natural type‐I hypolithon samples, assumed to be mature, were also sampled, in triplicate, from the same locations as the arrays. Adherent biomass was removed from the ventral surfaces of quartz rocks, embedded in the desert pavements, into Whirl‐Pak® bags. Samples were stored at 4°C immediately after collection and subsequently shipped on ice to the laboratory facilities at the Centre for Microbial Ecology and Genomics at the University of Pretoria, Pretoria, South Africa and stored at −80°C until extraction in 2021 and 2022.

### DNA extraction and sequencing

DNA was extracted from 0.75 g of soil using the QIAGEN DNeasy PowerSoil kit (Qiagen, Venlo, Netherlands). The protocol was modified to include an additional step of soil agitation using two 40 s cycles of 2500 rpm in a Powerlyzer 24 (Qiagen, Venlo, Netherlands). The quantity and quality of the DNA was evaluated with a Nanodrop 2000 spectrophotometer (Thermo Fisher Scientific, Waltham, United States). Negative control extractions were performed using sterile, distilled water.

The extracted DNA was submitted to Omega Bioservices (Norcross, Georgia, United States) for sequencing of the V3‐V4 region of the 16S rRNA gene (Forward primer: 5′‐CCTACGGGNGGCWGCAG‐3′, reverse primer: 5′‐GACTACHVGGGTATCTAATCC‐3′) (Klindworth et al., 2013) on an Illumina MiSeq v3 with 300 bp paired‐end reads.

### Data pre‐processing

The raw sequencing reads were processed in QIIME2 2020.8 (Bolyen et al., 2019) and denoised with DADA2 (Callahan et al., 2016). To remove poor‐quality bases and enhance pairing, reads were truncated to 280 and 220 bp for forward and reverse reads, respectively, and 15 bp were trimmed from the front of all reads. The resulting amplicon sequence variants (ASVs) were assigned taxonomically by comparing the sequences to the SILVA 138.1 (Quast et al., 2013; Yilmaz et al., 2014) database of 16S rRNA genes using a naive Bayes classifier.

To address potential sample contamination, we used Decontam (Davis et al., 2018), which removes taxa that display signals of contamination; that is, taxa whose relative abundance increases as the library concentration decreases, which removed 303 taxa. In addition, ASVs with fewer than 63 reads were filtered out in order to minimize false positives from the sequencing run. Filtering low‐abundance taxa has been shown to increase reproducibility while maintaining the structure of the data (Cao et al., 2021). After quality control, merging, and filtering, 8326 ASVs remained for analysis.

### Data analysis

Data were analysed in R 4.1.1 (R Core Team, 2021) using the following packages and their dependencies: phyloseq 1.36.0 (McMurdie & Holmes, 2013), decontam 1.12.0 (Davis et al., 2018), vegan 2.5‐7 (Oksanen et al., 2019), stringr 1.4.0 (Wickham, 2019), ggplot2 3.3.5 (Wickham, 2016), ggpubfigs 0.0.1 (Steenwyk, 2021), ggrepel 0.9.1 (Slowikowski, 2021), RColorBrewer 1.1‐2 (Neuwirth, 2014), gridExtra 2.3 (Auguie, 2017), rnaturalearth 0.1.0 (South, 2017), ggspatial 1.1.5 (Dunnington, 2021), sf 1.0‐3 (Pebesma, 2018), cluster 2.1.2 (Maechler et al., 2021), NbClust 3.0 (Charrad et al., 2014), ggdendro 0.1.22 (de Vries & Ripley, 2020), ggdendroplot 0.1.0 (Huber, 2021), ANCOMBC 1.2.2 (Lin & Peddada, 2020a), pheatmap 1.0.12 (Kolde, 2019), VennDiagram 1.6.20 (Chen, 2018), picante 1.8.2 (Kembel et al., 2010), dplyr 1.0.7 (Wickham et al., 2021).

#### Normalization

ASV counts were normalized using relative abundance/proportions for most analyses. While proportions are unreliable for detecting differential abundance, normalizing library sizes with rarefaction or proportion provide the most accurate comparisons between microbial communities (McKnight et al., 2019).

#### Alpha and beta diversity

Alpha diversity was calculated both as the number of observed ASVs and the Shannon index. Beta diversity was calculated using the quantitative/weighted Jaccard metric (Chen et al., 2021).

#### Differential abundance of taxa

To identify taxa that were differentially abundant, we used ANCOM‐BC (Lin & Peddada, 2020a), which has been consistently identified as a reliable method for detecting differential abundances (Lin & Peddada, 2020b; Nearing et al., 2021; Weiss et al., 2017). Differentially abundant ASVs were determined at the genus level. As the relative abundance of different ASVs can differ by orders of magnitude, each ASV abundance was scaled individually to aid in visualizing changes in ASV abundance between clusters and over time.

## RESULTS

### Visual evidence for the temporal development of de novo hypolithons

In this study, two arrays were established at different sites along a precipitation gradient, characterized by a fog‐inundated coastal zone, a hyper‐arid zone 60 km from the coast and a ‘higher rainfall’ zone approximately 120–280 km from the coast (Lancaster et al., 1984). The inland array was established in an area estimated to receive between 100 and 150 mm of annual rainfall (Bosch et al., 2022), while the ‘station’ array was positioned near the Gobabeb‐Namib Research Institute facilities, located within the central hyper‐arid zone.

The natural quartz pebbles allowed the transmission of between 1.1% and 25.4% of the incident light, a variation which would depend on the structure and purity of the quartz (see Cowan et al., 2011), while the cut marble tiles allowed for the transmission of 1.2%–5.6% of incident light. By comparison, travertine is 100% light opaque, and was therefore used as a ‘dark rock’ control. The percentage of transmission between the different rock types was significantly different (Kruskal‐Wallis test chi‐squared = 163.56, *p* < 2.2e‐16), with quartz rocks exhibiting the highest mean transmission percentage (Figure S2).

**FIGURE 2:**
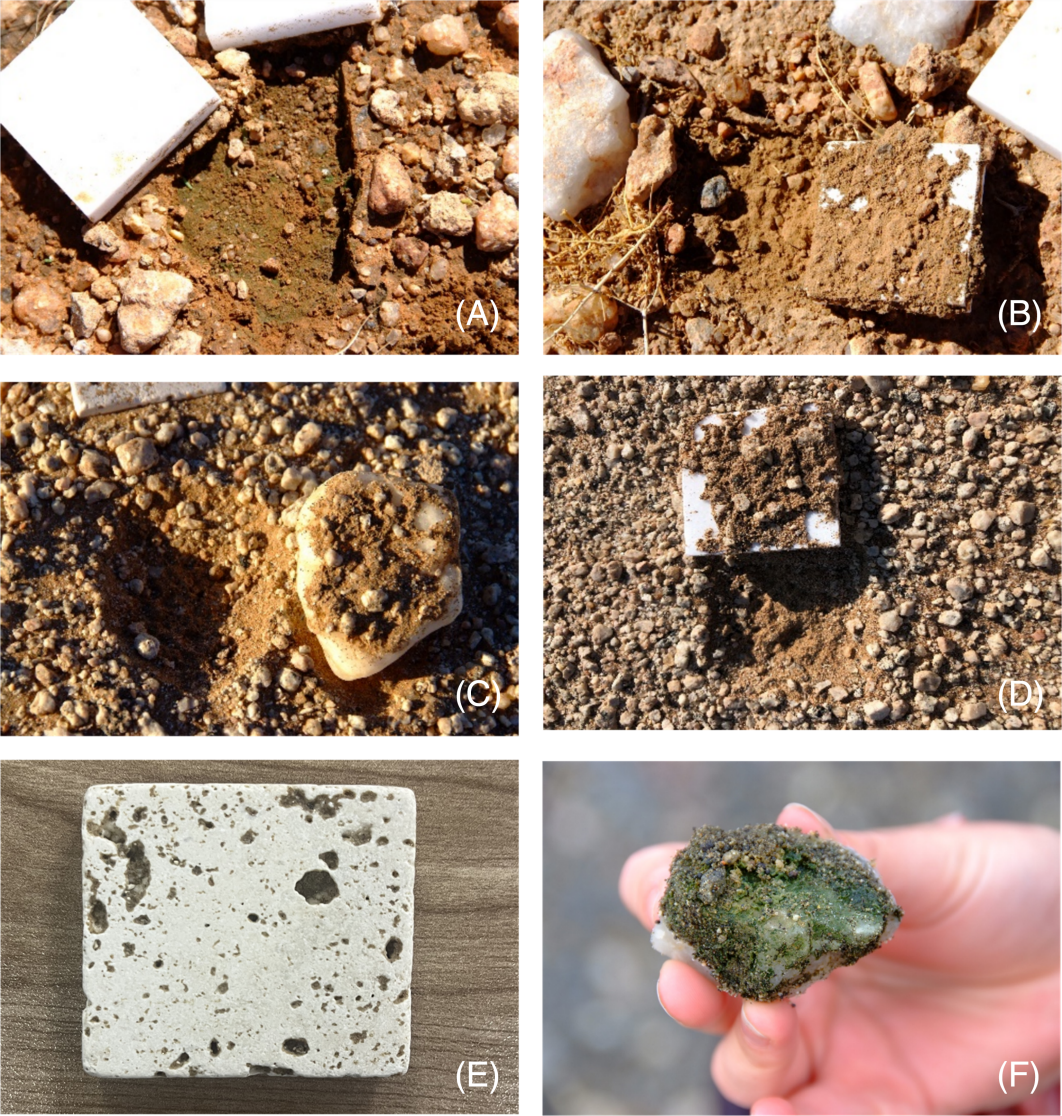
(A) Cyanobacterial biofilm on soil surface below quartz rock (removed) [April 2017]. (B) Adherent material on the ventral surface of an upturned marble tile [April 2018]. (C) Adherent material on the ventral surface of an upturned quartz rock [2019]. (D) Adherent material on the ventral surface of an upturned marble tile [2020]. (E) Example of a travertine tile, without adherent material, extracted from the inland array [2020]. (F) Example of a mature cyanobacterial biomass‐dominated hypolithon commonly found in desert pavements.

At the inland array, the first visual evidence of cyanobacteria‐dominated biofilm development on the soil surface beneath quartz and marble tiles was observed after 2 years (i.e., in 2017) (Figure 2A), but with no evidence of adherent material on the ventral surface of the tiles. For both marble tiles and quartz rocks, evidence of possible microbial colonization on the ventral surfaces, in the form of adherent material, was observed from year 3 onward (Figure 2B–D), while there was no evidence at any point of sampling of colonization on the ventral surfaces of non‐translucent travertine tiles (Figure 2E). However, the communities attached to the marble and quartz rocks had a morphology distinct from the natural hypolithons collected from the surrounding desert pavement (Figure 2F). No evidence of biofilm or hypolithon development was observed in the Station array at any sampling point.

### The microbial community structure of developing hypolithons

A total of 21 known phyla were identified from the 16S rRNA sequencing data from inland array samples, 11 of which could be considered as dominant phyla (accounting for more than 1% relative abundance) across the sample set (Figure S3). The five most abundant phyla were Cyanobacteria (26.5% +/− 15.7%), Pseudomonadota (20.0% +/− 4.4%), Actinobacteriota (13.3% +/− 3.2%), Chloroflexota (8.4% +/− 2.3%), and Bacteroidota (7.1% +/− 3.0%). Three rare phyla (abundance below 1% of total reads), Armatimonadota, Patescibacteria, and Deinococcota, were also found to be ubiquitous across samples, consistent with their widespread distribution in top‐soil environments (Beam et al., 2020; Dose et al., 2001; Lee et al., 2014).

The measured alpha diversity (Figure S4) was highly variable across rock types and years. Although the trends were not statistically significant, we observed that the hypolithons of quartz and marble tiles were generally less rich and more uneven (i.e., dominated by fewer taxa) than those of the control soil and travertine tiles. Our confidence in this trend is strengthened by the fact that mature, natural hypolithons showed the lowest levels of diversity; suggesting that the decreasing richness and evenness was a sign of maturing hypolithons. The large variation in the quartz and marble hypolithon diversities shows that 7 years is insufficient time for the development of the hypolithic community to full maturity.

**FIGURE 3:**
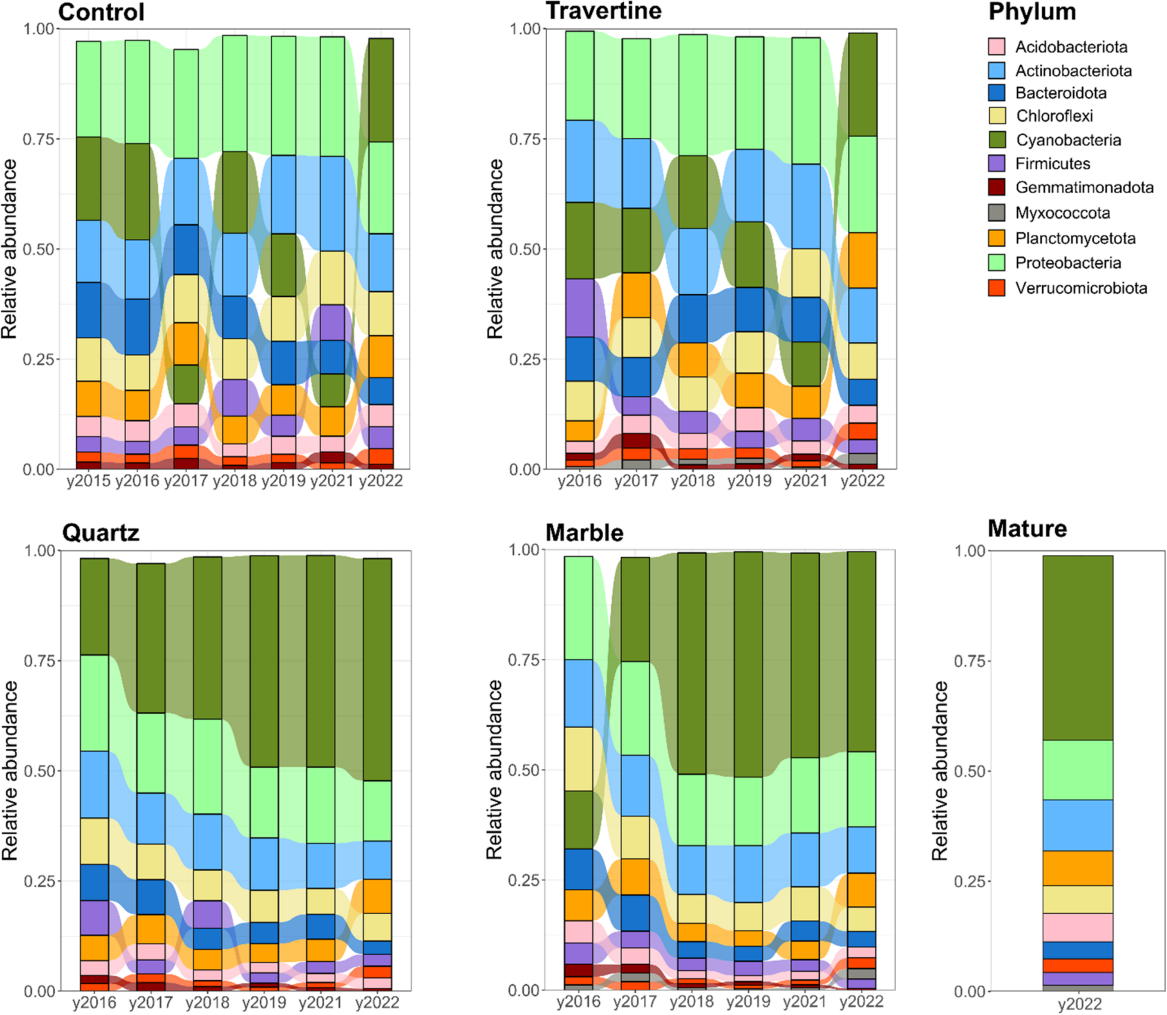
Alluvial plots showing the shift in relative abundances of dominant phyla in inland array sample sets across different years. The relative abundance of dominant phyla are expressed as the average of the triplicate samples taken for each year (2015–2022). Dominant phyla represent those phyla that account for more than 1% of total reads across the sample set for each rock type and year. Relative abundance is expressed as the fraction of total reads, in a scale from 0 to 1. In barplots for each year, phyla are arranged in decreasing order of abundance, from the phylum with the highest relative abundance on top, to the phylum with the lowest relative abundance. Samples were clustered according to the following nomenclature: Control – samples collected from open soil; Travertine – samples taken from soil under travertine rocks; Quartz – samples collected from ventral surface of the quartz rocks and from soil underneath; Marble – samples collected from the ventral surfaces of the marble rocks, and the soil underneath; Mature – Samples collected from the ventral surfaces of quartz rocks occurring naturally at the site of the inland array.

These results are mirrored by observed shifts in the abundance of dominant phyla in marble and quartz tile samples from 2017 onwards (Figure 3). These shifts were mainly reflected by a significant increase (*p*‐value <0.05) in Cyanobacteria (hence referred to as a cyanobacterial ‘bloom’), followed by a significant decrease in the fraction of Pseudomonadota and Bacteroidota (Figure S5).

By comparison, no significant changes in the relative abundance of the dominant phyla were detected in the travertine and soil control sample groups across the years, supporting the visual observations that hypolithon‐like communities do not develop under non‐translucent rocks or in surface open soils, and the obvious conclusion that hypolithon development is light‐driven. We note that in the 2022 samples, an increase in the abundance of Cyanobacteria was also observed in both the soil control and travertine groups. We suggest that a significant rainfall in April 2022, just prior to sample collection, drove an increase in the soil cyanobacterial population.

**FIGURE 4:**
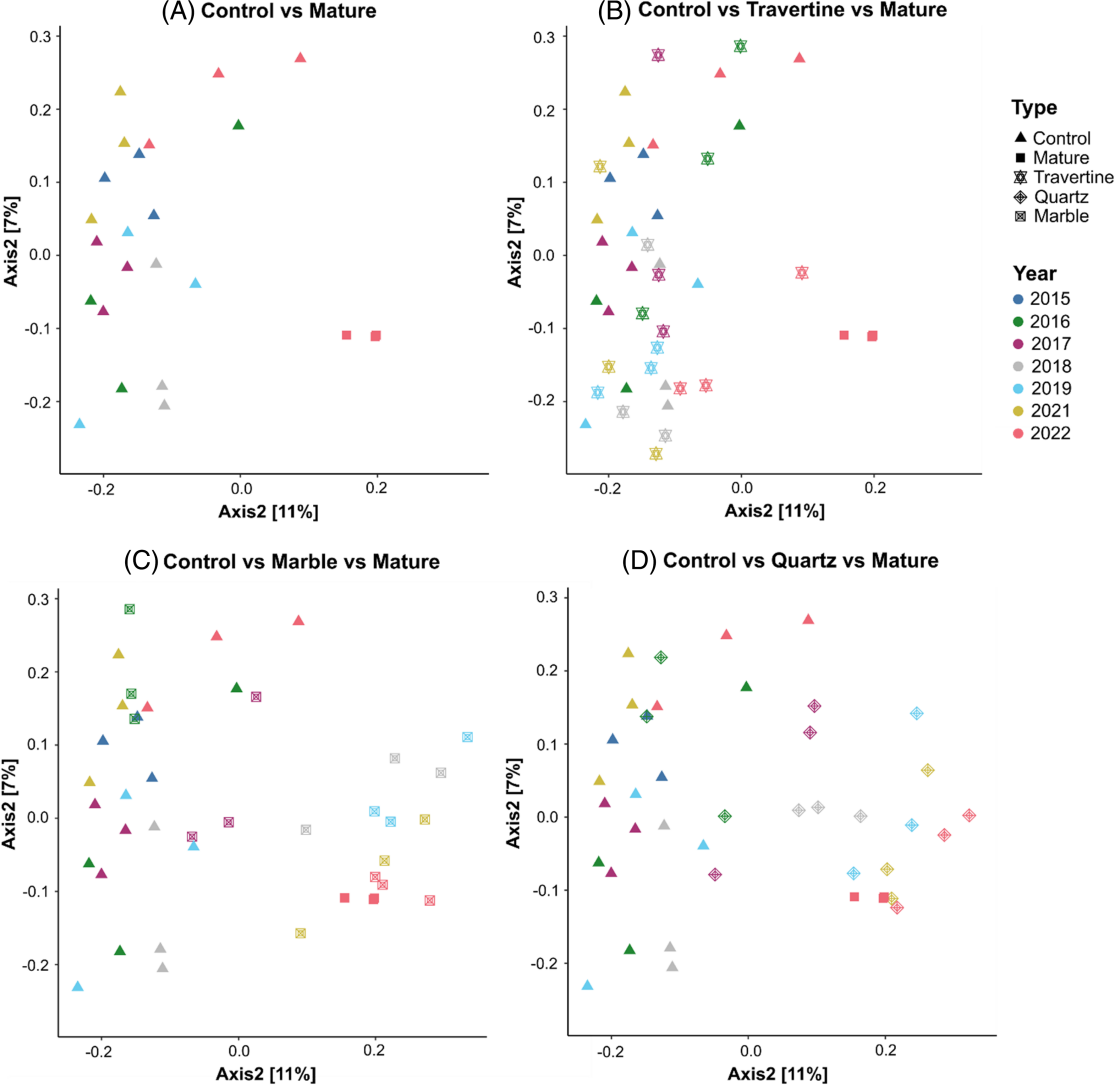
Community structure distribution of samples across different rock types and year. The PCoA plots show the distribution of the beta‐diversity (Jaccard distance) of samples on a 2‐dimension ordination plane. The four panels represent the comparison between different sets of samples: (A) Control versus Mature; (B) Control versus Travertine versus Mature; (C) Control versus Marble versus Mature; (D) Control versus Quartz versus Mature. Samples are coloured according to year (2015–2022), while sample shapes vary according to type of rock (Soil Control, Marble, Quartz, and Travertine).

The shift in microbial composition inferred by the differential abundance of dominant phyla was also observed in the overall microbial community structures of the samples (as expressed by Jaccard beta‐diversity), which was significantly related to two different variables; the rock type and the year (Figure 4). We observed that both variables, including the interaction between them, explained a significant proportion of the variation of the microbial community when tested by PERMANOVA (rock type: *R* ^2^ = 0.11, *p* < 0.001, year: *R* ^2^ = 0.13, *p* < 0.001, type X year: *R* ^2^ = 0.20, *p* < 0.001). These differences in community structure between samples were reflected in an increased percentage of total shared ASVs between marble and quartz tile samples, driven by the substantial increase in shared cyanobacterial ASVs (Figure S6). In turn, this increase is mirrored by a general decrease in total shared ASVs between these two groups and the soil control and travertine samples, which exhibited much lower percentages of shared cyanobacterial ASVs.

**FIGURE 5:**
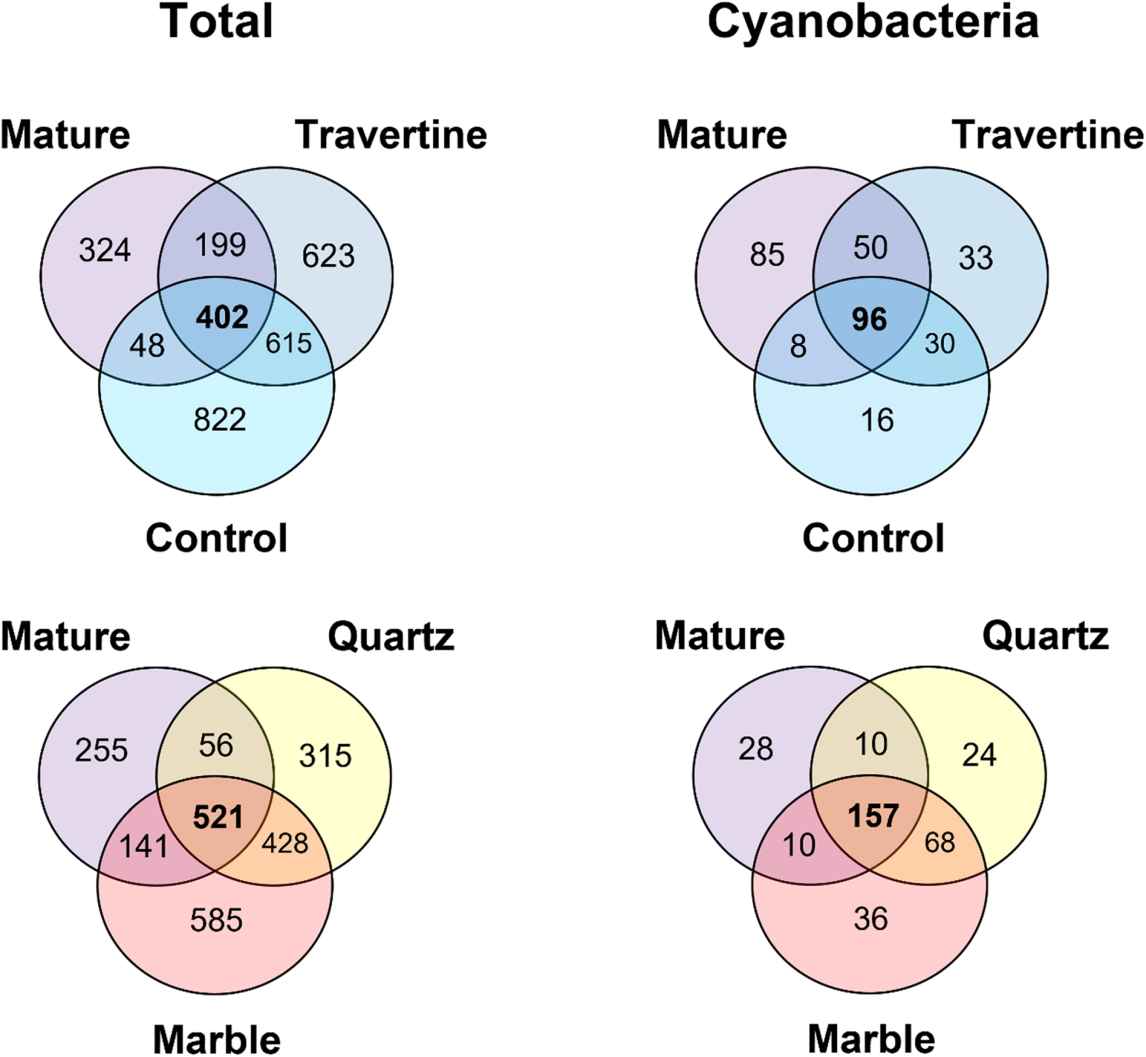
Venn diagram showing the number of shared ASVs between sample type, across the 2022 inland array dataset. Both total number of shared ASVs and number of shared Cyanobacteria ASVs were calculated.

Both the alpha‐ and beta‐diversity analyses indicated that by 2022, microbial communities growing under quartz rocks and marble tiles exhibited some similarities in biodiversity and community structures as mature type‐I hypolithons existing in the same environment. Notably, quartz and marble tile samples were shown to share more ASVs with mature hypolithons than soil control or travertine samples (Figure 5). It is worth noting however that while the mature, quartz and marble communities share the majority of cyanobacterial ASVs, there is still a large number of ASVs that are unique to each group, highlighting the point that the communities under the quartz and marble tiles sampled in 2022 are not representative of mature hypolithon communities found in the desert pavement.

### Differential abundance of taxa between different rock types

**FIGURE 6:**
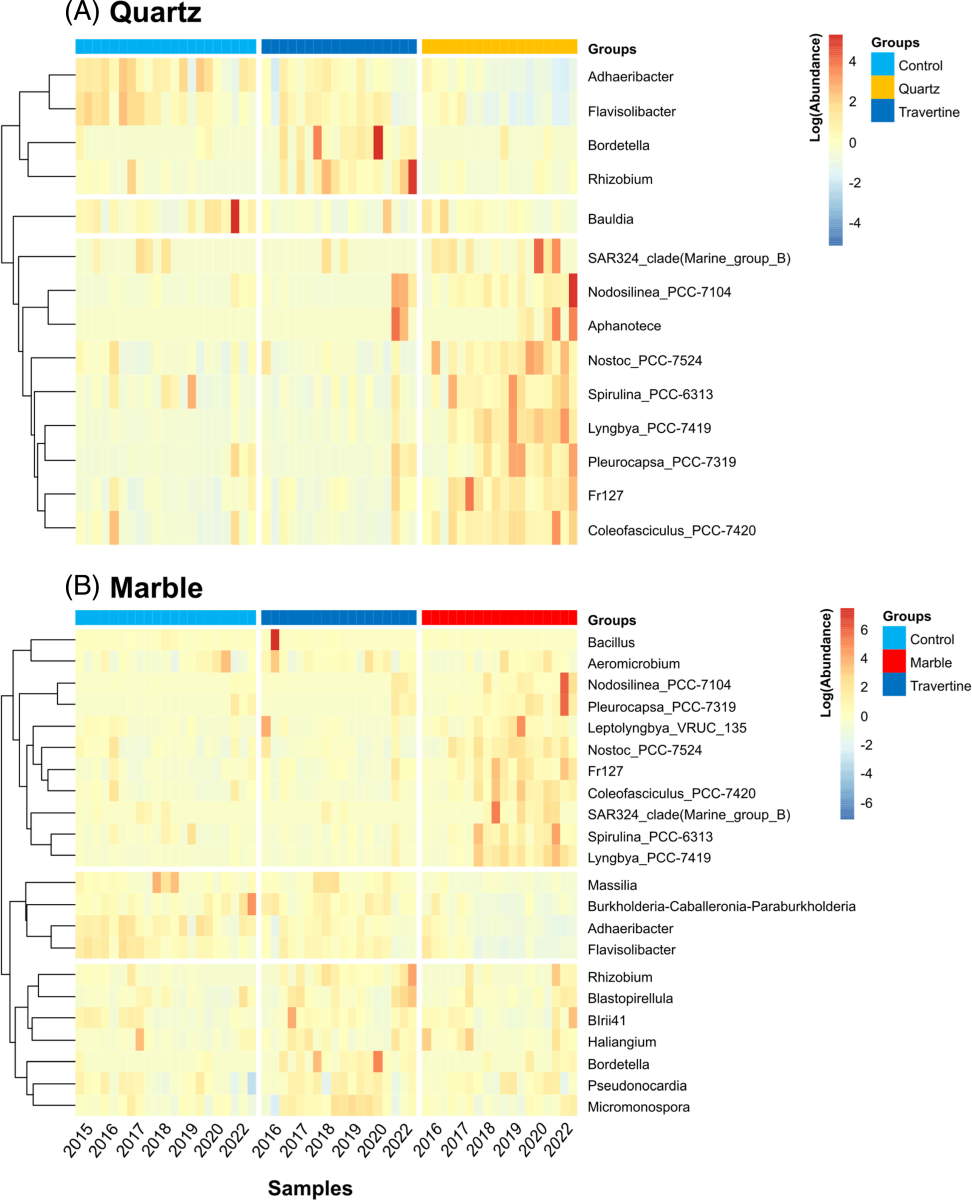
Differentially‐abundant taxa between sample groups in the inland array. This analysis was done for both Quartz (A) and Marble (B) sample groups, which were compared against the Soil Control/Travertine samples. Each column represents one sample with colours indicating the sample groups. Columns were ordered from left to right according to year of sampling for each rock type. Each row represents one genus‐level ASV which was identified as being differentially abundant between the sample clusters. The genus‐level ASVs were clustered using Euclidean distances. Relative abundances were scaled and centred per genus‐level ASV which allows for comparison between ASVs whose absolute abundance may vary through orders of magnitude. Only taxa with a known genus classification were represented in the heatmaps.

To understand the compositional development of the hypolithic microbial communities at the genus level, ANCOM‐BC was used to identify differentially abundant genera found in quartz rock and marble tile derived samples compared to samples where no hypolithon development was detected. A total of 28 and 36 differently abundant genera were detected in quartz and marble samples compared to travertine and control samples (Figure 6A, B), respectively. The majority of over‐represented genera in both the quartz and marble samples belonged to the Cyanobacteria phylum (94.7% and 94.5% of over‐represented genera, respectively), with the most over‐represented known genera being *Coleofasciculus PCC‐7420* (5.3% and 5.8% average relative abundance for quartz and marble, respectively) and *Lyngbya PCC‐7419* (3.5% and 3.1% average relative abundance for quartz and marble, respectively). Other Cyanobacterial families that have been previously identified as highly prevalent in hypolithons, such as *Phormidiaceae* (Caruso et al., 2011; Lacap et al., 2011; Lacap‐Bugler et al., 2017; Van Goethem et al., 2017), were also present in developing quartz rock and marble tile hypolithon communities, but at less than 1% relative abundance, suggesting that members of these Cyanobacterial families are not primary colonizers.

In an analysis of the abundance of the top six known cyanobacterial genera over‐represented in quartz and marble tile samples over time (Figure S7), we observed distinctly different development dynamics of the various taxa. For instance, *Lyngbya PCC‐7419* exhibited a steady and significant increase of relative abundance in both quartz and marble rock communities with time. In marble tile samples, *Lyngbya* PCC‐7419 increased sharply in 2018, followed by a steady decrease in subsequent years, but still with significantly higher relative abundances than in control and travertine samples. Similarly, the relative abundance of *Coleofasciculus PCC‐7420* increased dramatically in marble tile samples at year 3, followed by a progressive decrease over time. Other taxa, such as *Nostoc PCC‐7524* and *Nodosilinea PCC‐7104*, exhibited broadly similar trends for the two translucent rock types, while *Pleurocapsa PCC7319* was shown to steadily increase in abundance with time but only in marble tile communities.

### Hypolithon development in different regions of the Namib Desert

To test if the kinetics of hypolithon development would be similar in the central hyper‐arid zone (compared with the ‘inland’ array in the rainfall zone), a second hypolith array was established in April 2015, near the Gobabeb‐Namib Research Institute in the hyper‐arid central region of the Namib Desert (the ‘Station’ array).

Comparisons of the microbial community structure of control soils between the two arrays (corresponding to *t* = 0) showed a significant clustering of samples by location (*R* ^*2*^ = 0.17, *p*‐value <0.01) (Figure S8), indicating that the open soils at the two array sites harbour distinct microbial communities (Scola et al., 2017). This was further confirmed by the low percentage of shared ASVs between arrays (from 2.8% to 6.2% of total ASVs), not just for the Soil Control samples (average 3.5% shared ASVs), but also for the different types of rock as well as the mature hypolithons collected at each array (5.7% shared total ASVs; 6.1% shared cyanobacterial ASVs) (Figure S9).

**FIGURE 7:**
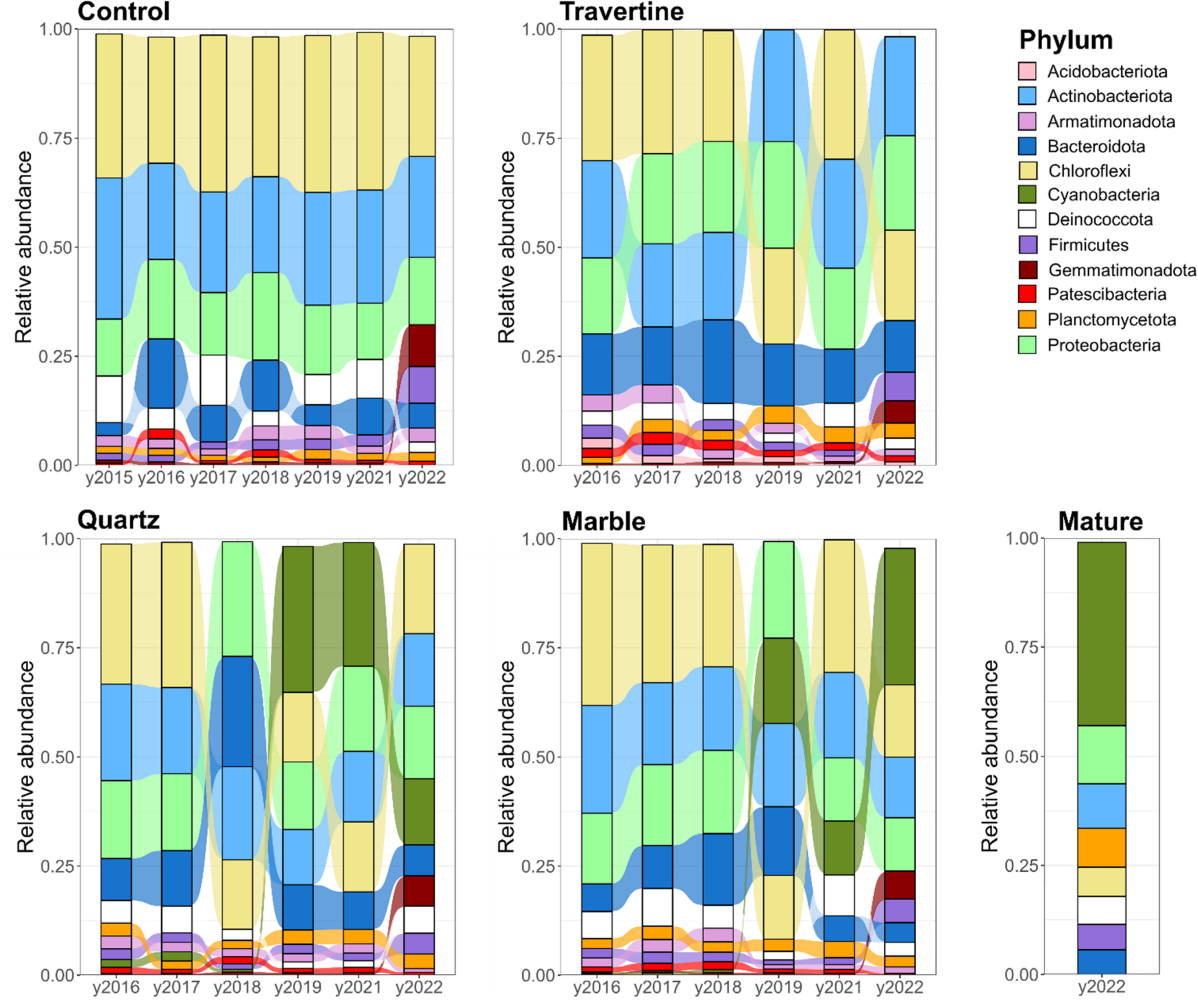
Alluvial plots showing the relative abundance of dominant phyla in samples across the station array. Samples were grouped according to rock type and year. Dominant phyla represent those phyla that account for more than 1% of total reads across the sample set. Relative abundance is expressed as the fraction of total reads. In barplots for each year, phyla are arranged in decreasing order of abundance, from the phylum with the highest relative abundance on top, to the phylum with the lowest relative abundance. Samples were clustered according to the following nomenclature: Control – samples collected from open soil; Travertine – samples taken from soil under travertine rocks; Quartz – samples collected from ventral surface of the quartz rocks and from soil underneath; Marble – samples collected from the ventral surfaces of the marble rocks, and the soil underneath; Mature – Samples collected from the ventral surfaces of quartz rocks occurring naturally at the site of the station array.

The most obvious differences between the two regional sites were the complete absence of cyanobacterial signatures in the Soil Control and Travertine tile samples from the Station array, and a dominance of the phyla Chloroflexota (30.4% average Rel. Abund.) Actinobacteriota (21.2%) and Pseudomonadota (0.17%) (Figure 7). Cyanobacteria were first detected at relative abundances above 1% in year 4 marble and quartz tile samples, suggesting that the growth rates of Cyanobacteria, even under protective translucent quartz substrates, are substantially lower in the hyper‐arid zone than in the wetter inland zone. Despite the increase in Cyanobacterial signals from 2019 onward, none of the samples recovered from the station array showed any visual evidence of adherent material on the ventral tile or rock surfaces, and there was no visual evidence of cyanobacterial biofilm development on the soil surfaces below the embedded tile and rock samples until the last year of sampling (2022).

**FIGURE 8:**
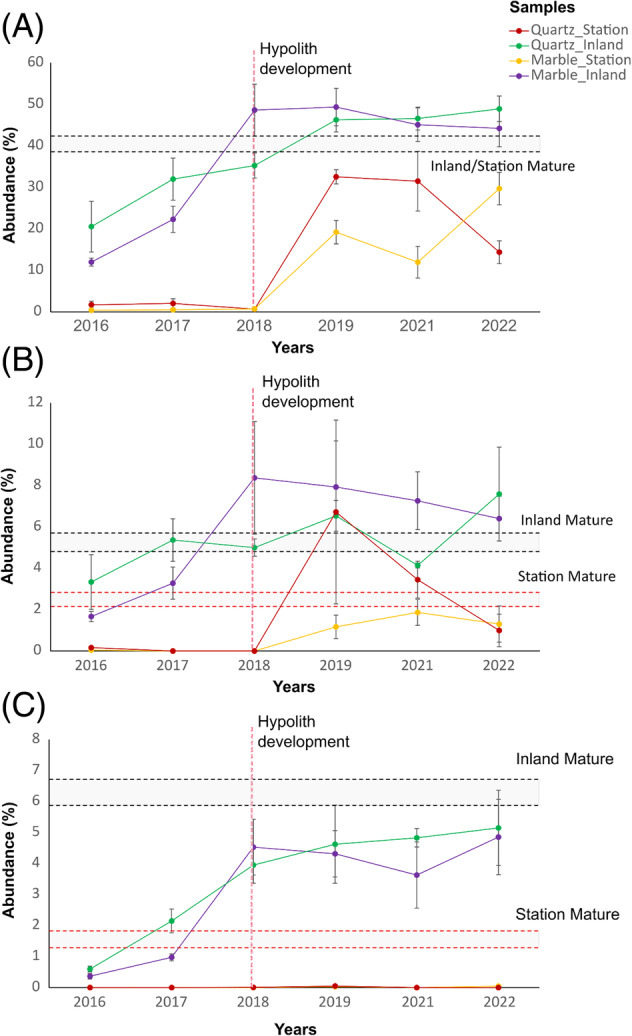
Comparison of the growth dynamics of Cyanobacteria between Marble and Quartz samples at the inland and station arrays. The line graphs represent the shifts in relative abundance (%) of the phylum Cyanobacteria (A), as well as the genera *Coleofasciculus* *PCC‐7420* (B) and *Lyngbya* *PCC‐7419* (C), as a function of sampling year, for Quartz and Marble samples in both station and inland arrays. The vertical dashed line represents the sample time point at which we obtained visual evidence of microbial attachment to the rocks at the inland array. Black and red dashed lines indicate the relative abundance ranges observed in mature hypoliths from inland and station arrays, respectively, for each represented taxon. In the case of the phylum Cyanobacteria, both the mature hypoliths of the inland and station arrays exhibited overlapping relative abundance ranges, and therefore were combined into a single range. The error margins for each time point were calculated as the standard deviation divided by the square root of the number of samples.

To investigate whether a ‘critical mass’ (relative abundance threshold) of cyanobacterial signatures was associated with the visual observations of initial hypolithon colonization, the dynamics of cyanobacterial growth over the time frame of the study were compared between marble and quartz tile samples at both arrays (Figure 8). Mature, natural hypolithons from both array sites exhibited similar average relative abundances of Cyanobacteria (40%), a threshold that was reached in both quartz and marble sample at the inland array by year 3 (Figure 8A) and which was consistent with the visual observations of hypolithon development at this site. By contrast, the abundance of Cyanobacteria in quartz and marble samples at the Station array never reached the same relative abundances throughout the study period. Furthermore, a similar trend was observed when performing the same analysis on the relative abundance of *Lyngbya PCC‐7419* and *Coleofasciculus PCC‐7420* (Figure 8B, C), which were identified as potential early ‘colonizers’ (Figure S7). Notably, the relative abundance of *Lyngbya PCC‐7419* in the quartz and marble tile samples at the inland array increased progressively over the study period, approaching the relative abundances found in mature hypoliths sampled in the vicinity of the array. No increases in relative abundance of the same taxa were detected in samples from quartz rocks and marble tiles in the Station array (Figure 8C).

## DISCUSSION

The Namib Desert is characterized by a very well established longitudinal water gradient, from the coast to the inland mountains (Eckardt et al., 2013; Lancaster et al., 1984). This gradient is nominally defined as three zones: (i) the arid coastal zone, subject to regular inundations of sea‐fog (Olivier, 1995), which extends from the coast to approximately 60 km inland, (ii) the central hyper‐arid zone, extending a further 60 km from the coast, and (iii) an inland ‘rainfall’ zone approximately 120–180 km from the coast (Lancaster et al., 1984). Quartz pebbles and their associated hypolithic communities occur across the entire transect (Warren‐Rhodes et al., 2013).

This study provides the first quantitative estimate of a timeline for initial hypolithon development. The visual evidence of chlorophyll‐containing biomass under the semi‐translucent tiles in the inland array at year 2, and subsequent evidence of ventral surface attachment at year 3 suggests that cyanobacterial‐driven biofilms under semi‐translucent rocks start developing within 36 months after deposition of the tiles on the soil surface. These soil surface biofilms are presumably the precursor for the subsequent attachment into the ventral sides of the translucent rocks. The fact that chlorophyll‐containing biomass was visually detected under semi‐translucent tiles in the Station array at the last year of sampling, despite the absence of visual evidence of attachment to the ventral rock surfaces, suggests that hypolithon development in the hyper‐arid zone preceded at a slower rate than in the rainfall zone. These results were confirmed by the significant increase in the relative abundance of cyanobacterial 16S rRNA gene sequences from year 2 onwards in the quartz and marble samples at the inland array, while an equivalent increase was only detected at year 7 at the Station array. The Cyanobacterial bloom detected in the biomass recovered from the ventral surfaces of both semi‐translucent quartz and marble rocks is strongly suggestive of the development of type I (cyanobacteria‐dominated: Cowan et al., 2010) hypolithon communities (Caruso et al., 2011; Cockell & Stokes, 2004; Lacap‐Bugler et al., 2017; Lebre et al., 2020; Van Goethem et al., 2017). Our results also suggest that, given appropriate conditions, hot desert type I hypolithon development can be initiated over relatively short periods of time (<3 years). The increase in the percentage of shared cyanobacterial ASVs between the quartz and marble tile sample communities as time progressed also suggests that hypolithon development in these two rock types might share common pathways of cyanobacterial ‘colonization’, despite the recorded differences in the amount of light transmission for the rock types. In turn, the absence of cyanobacterial enrichment or visual hypolithon development on the ventral surfaces of opaque rocks is consistent with the concept that the light‐transmissive properties of the over‐lying mineral substrate are essential for photosynthesis‐driven hypolithon development (Gwizdala et al., 2021).

During the development of the hypolithic communities, we observed and inverse relationship in the relative abundances of Cyanobacteria and Bacteroidota/Pseudomonadota, which might be explained by the trophic relationships between these phyla. In open surface soils, phototrophic activity is likely to be very low due to the extended periods of very low water activity (Bosch et al., 2022), consistent with the low abundance of Cyanobacteria (León‐Sobrino et al., 2019). The fact that trace gas (H_2_/CO) chemotrophy has been suggested as the primary energy acquisition process for community subsistence and growth in desert soils may be particularly significant (Jordaan et al., 2020; Ortiz et al., 2021). Both Pseudomonadota and Bacteroidota have been shown to be important taxonomic groups in trace gas chemotrophy (Bay et al., 2021; Ortiz et al., 2021). Therefore, the increase in relative abundance of Cyanobacteria and corresponding decrease in relative abundances of Bacteroidota/Pseudomonadota in communities under quartz and marble samples might indicate a shift in trophic strategy, during hypolithon development, from trace gas chemotrophy to phototrophy.

It is also important to note that despite some similarities in microbial composition (indicated from the alpha‐ and beta‐diversity metrics, as well as number of shared ASVs) between samples from the quartz/marble tiles and mature hypolithons, the latter were still highly distinct. Given that mature hypolithons are estimated to be hundreds, and possibly thousands, of years old (Bonani et al., 1988; Warren‐Rhodes et al., 2006), our data can only be interpreted in terms of the initiation of hypolithic community development. We suggest, therefore, that the adherent soil recovered from the ventral surfaces of quartz rocks and marble tiles from 2017 onward represent early stages in the development of mature hypolithons (Pointing & Belnap, 2012). Certainly, the adherent soil was visually distinct from the thallus‐like chlorophyll pigmented cyanobacterial biomass typical of a mature hypolithon.

The two most over‐represented genera in samples from both the quartz and marble tiles in the inland array, *Coleofasciculus PCC‐7420* and *Lyngbya PCC‐7419*, probably play important roles in hypolithon formation. *Coleofasciculus PCC‐7420* corresponds to the taxon *Microcoleus chthonoplastes*, which was renamed in 2008 (Siegesmund et al., 2008). This filamentous cyanobacterium is found in microbial mats in many marine and soil environments (Garcia‐Pichel et al., 1996; Prufert‐Bebout & Garcia‐Pichel, 1994), and is recognized by the formation of trichomes, multicellular structures that may assist with light capturing and protection against UV‐radiation (Tamulonis et al., 2011). These specialized structures are a common feature in biocrusts (Cano‐Díaz et al., 2018) and may therefore play a role in the formation of the hypolithic biofilms (Kvíderová et al., 2018). Similarly, the genus *Lyngbya* may be important in the stabilization of the hypolithon structure due to its filamentous morphology (Jones et al., 2011; Onodera et al., 1997). We can speculate that the increased presence *Lyngbya* *sp.* in the communities of quartz and marble tile samples might also be associated with the observed reduction of community diversity over time, as several *Lyngbya* *sp.* strains have been shown to produce a broad range of antimicrobial compounds (Kumar et al., 2014; Zainuddin et al., 2009). The importance of these two taxa was further suggested by the dramatic increase of their abundance in the early stages of hypolithon development, which suggests a possible role as primary ‘colonizers’.

It has been frequently suggested that the rate of hypolithon development is strongly influenced by the extent of liquid water availability (Pointing & Belnap, 2012; Ramond et al., 2018; Warren‐Rhodes et al., 2013), although we note that other abiotic (e.g., mean annual temperature, soil chemistry, soil nutrient status) or biotic factors (e.g., the presence or number of precursor taxa) might also be significant drivers. Several studies have documented the dependence of type I hypolithon development on water availability, principally due to the water requirements for Cyanobacteria‐driven photosynthesis (Bosch et al., 2021; Gwizdala et al., 2021; Warren‐Rhodes et al., 2006; Warren‐Rhodes et al., 2013). The markedly lower relative abundances of Cyanobacterial taxa at the quartz and marble samples of the Station array compared to the Inland array, as well as the lack of visual observations of hypolithon development on the ventral sides of the tiles in this array, suggest that the lower water input regime of the hyper‐arid region (where the Station array was situated) has a large negative impact on the kinetics of cyanobacterial growth and, by extension, hypolithon development. In fact, the results from this study indicate that a threshold of cyanobacterial growth (i.e., the cyanobacterial ‘bloom’ recorded in the Inland array) might be a crucial step for the attachment of the Cyanobacteria biofilms to the ventral sides of semi‐translucent rocks. In addition, the absence of increase in the relative abundance of *Lyngbya* *PCC‐7419* in the Station array further points to a potential role of this taxa in the initial colonization processes leading to hypolithon development.

## CONCLUSIONS

In this study, we provide the first estimate of the kinetics of in situ hypolithic community development over time in hot desert pavements. Taken together, our data suggest that the pathway of hypolithic community development is triggered by recruitment of specific taxa (particularly Cyanobacteria) from the underlying soil to initially form a biofilm on the soil surface below the translucent rock (the ‘biofilm phase’). In wetter regions (the ‘Inland’ array site), this process occurs within 48 months, while in drier regions (the ‘Station’ array site) the first evidence of this process was only observed after 7 years. Within a further 12 months at the ‘Inland’ array site, there was evidence of a second stage of hypolithon development, where we observed biomass‐rich soil particles adhering to the ventral surfaces of translucent rocks (the ‘adherent phase’: Figure 2), a normal characteristic of mature hypolithons (Chan et al., 2012; Cowan et al., 2010). Up to the termination of the sampling program (7 years; 84 months), the hyper‐arid ‘station’ array showed no evidence of the second stage of hypolithon development (i.e., no adherence to the mineral surface).

Our data also suggest that the kinetics and pathways for hypolithon development are distinct between areas with different precipitation regimes. In the hyper‐arid zone of the Namib Desert, extreme desiccation is likely to affect the rates of cyanobacterial growth by restricting the metabolic window in which Cyanobacteria can photosynthesise (Bosch et al., 2021), which in turn will extend the time frame for initial colonization of the rock substrate, as observed by the late onset and low abundance of cyanobacterial signatures in the quartz and marble tile samples at the Station array. Dispersal limitations and local recruitment have also been previously suggested as important stochastic drivers of hypolithon development (Lebre et al., 2021; Makhalanyane et al., 2013), and in this study we present evidence of how differences in the extant microbial populations of open soils at the Inland and Station array sites might have affected the developmental pathways of hypolithons in these areas. In parallel, water availability probably plays both a direct and indirect role in shaping hypolithon development, by shaping the local soil microbial population and driving the rates of cyanobacterial growth.

The transition from ‘biofilm phase’ to ‘adherent phase’ clearly involves a mechanism of attachment (of soil particles and biomass to the mineral surface). We suggest that this process may be driven by the cellular production of extracellular polysaccharides (EPS), the insoluble complex carbohydrate matrix that is the structural basis for many microbial biofilms (Di Martino, 2018; Flemming et al., 2007), including those of mature hypolithons (De Los Ríos et al., 2014). The production of EPS is known to be triggered by stress conditions, particularly desiccation stress (Lebre et al., 2017). We therefore suggest that repeated wet‐dry cycles in desert soil, involving saturation and desiccation, may be the driver for EPS production in sub‐lithic biofilm communities, generating the adhesive components necessary to produce adherent structures typical of mature hypolithons.

We argue that the hypolithic communities observed under semi‐translucent quartz and marble tiles in the ‘Inland’ array are very far from mature communities, and that a ‘third stage’ of community development is necessary for the transition from soil‐dominated precursor communities to mature cyanobacterial biomass‐dominated communities. The time‐scales of the third developmental stage (from precursor to mature) remain unknown, but could potentially be measured in decades.

**FIGURE 9:**
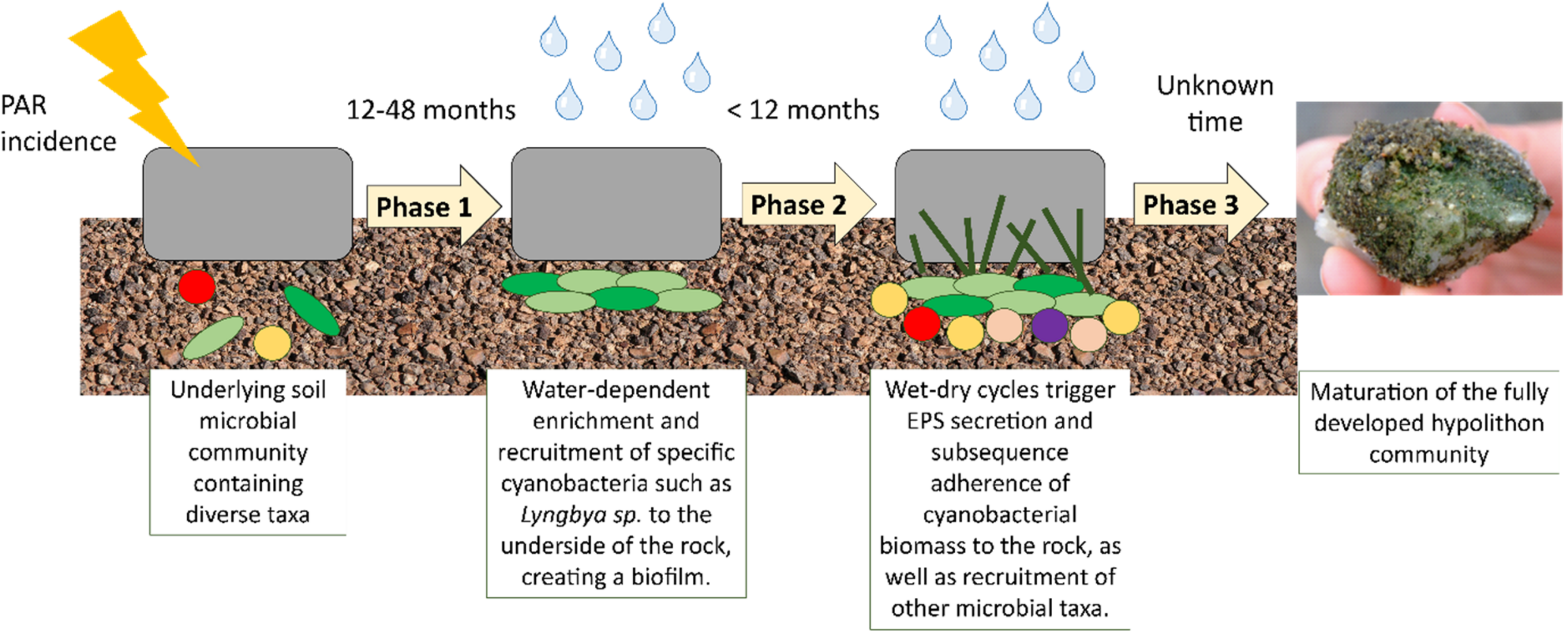
General pathway for hypolithon development in hot deserts. This conceptual schematic represents our proposed generalized pathway and timeline for the development of hypolithons in the pavements of the Namib Desert, taking into account the main abiotic factors that drive this development.

Based on this study, we propose a pathway of hypolithon development (Figure 9) involving initial recruitment of soil Cyanobacteria to the illuminated soil surface immediately underneath a translucent rock (Phase 1), a transition to an adherent state, possibly driven by cellular EPS production triggered by wet‐dry cycles (Phase 2), and a further maturation of the adherent community to a cyanobacterial biomass‐dominated mature community (Phase 3).

## AUTHOR CONTRIBUTIONS

**Jason Bosch:** Data curation (equal); formal analysis (equal); investigation (equal); methodology (equal); validation (equal); visualization (supporting); writing – original draft (lead); writing – review and editing (supporting). **Pedro H. Lebre:** Data curation (equal); formal analysis (equal); investigation (equal); methodology (equal); resources (lead); validation (equal); visualization (lead); writing – original draft (supporting); writing – review and editing (lead). **Eugene Marais:** Resources (supporting); validation (supporting); writing – review and editing (supporting). **Gillian Maggs‐Kölling:** Resources (supporting); validation (supporting); writing – review and editing (supporting). **Don A. Cowan:** Conceptualization (lead); funding acquisition (lead); investigation (supporting); methodology (equal); project administration (lead); resources (equal); supervision (lead); validation (equal); writing – review and editing (equal).

## CONFLICT OF INTEREST STATEMENT

The authors declare no conflicts of interest.

## Supporting information


**Data S1.** Supporting information.

## Data Availability

The raw sequencing data has been deposited to the National Center for Biotechnology Information (NCBI) Sequence Read Archive (SRA) as BioProject PRJNA1051043.
