# Supplementary material for: Kinetics and pathways of sub‐lithic microbial community (hypolithon) development
Source: Environ Microbiol Rep. 2024 Jun 23;16(3):e13290. doi: 10.1111/1758-2229.13290 (PMC11194044; doi:10.1111/1758-2229.13290)
Supplement: Supplementary file 1 — Data S1. Supporting information. [file EMI4-16-e13290-s001.docx]

**APPENDIX**


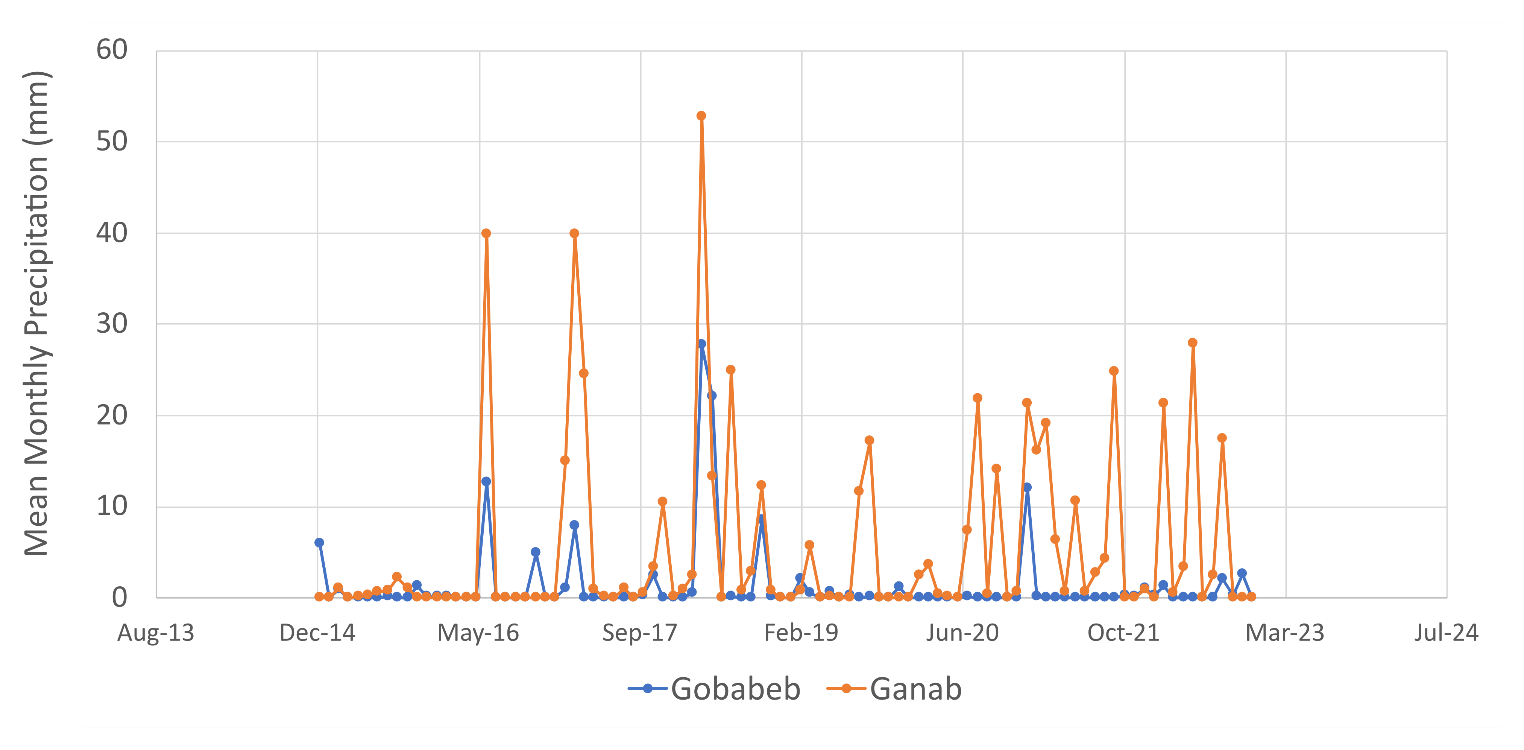


**Figure S1. Mean monthly precipitation (mm) recorded at meteorological stations close to the array sites, over a period between Jan 2015 and Dec 2022.** The Gobabeb meteorological station is located at the same site as the station array, while the Ganab station is located 28 km from the inland site.


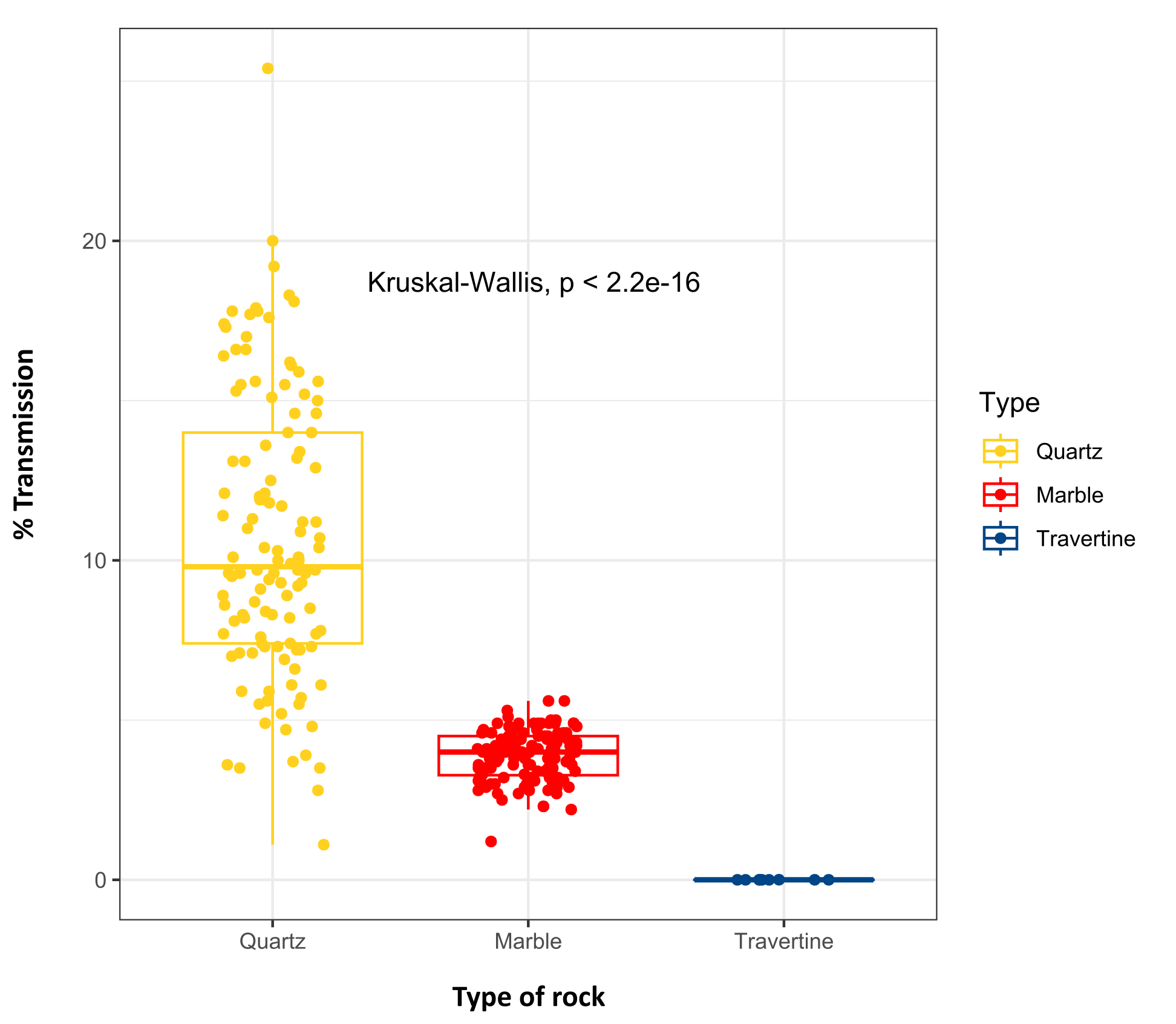


**Figure S2.** **Boxplot showing the percentage of transmission for the rock pebbles used in the arrays, grouped by rock type.** A total of 249 measurements were made (120 for quartz; 120 for marble; 9 for travertine). Significance of the differences between the three rock types was calculated using the Kruskal-Wallis test, taking into consideration that the data was non-normally distributed.


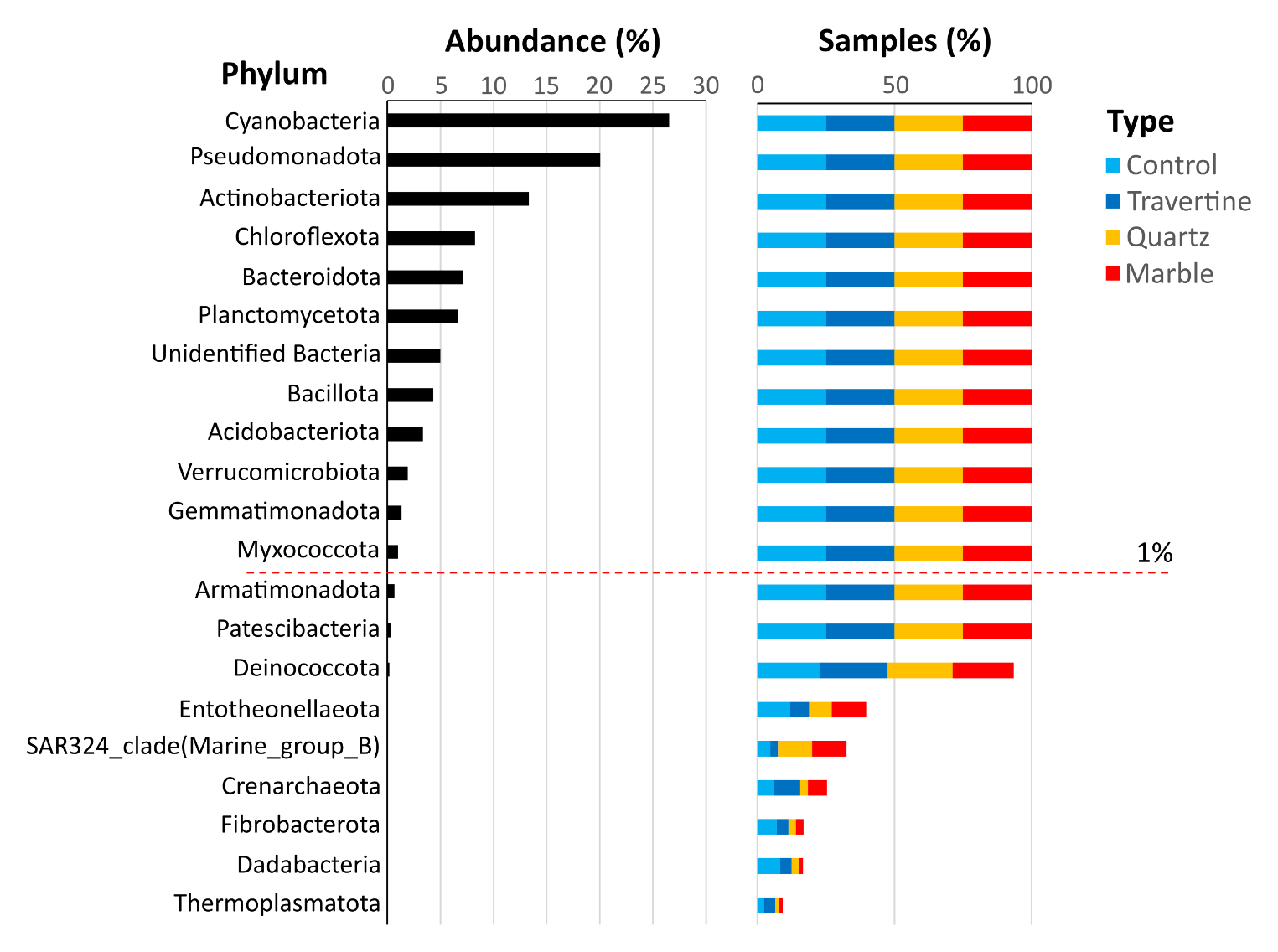


**Figure S3. Abundance and distribution of phyla across the inland dataset.** Phyla are ordered in terms of relative abundance, which is expressed as the average relative abundance (%) across all samples in the dataset. The red dashed line represents the cut-off above which phyla are considered dominant in the community. Distribution was broken down by type of sample, and is expressed as the percentage of samples containing the phyla within each group, with 25% representing the total number of samples for each group.


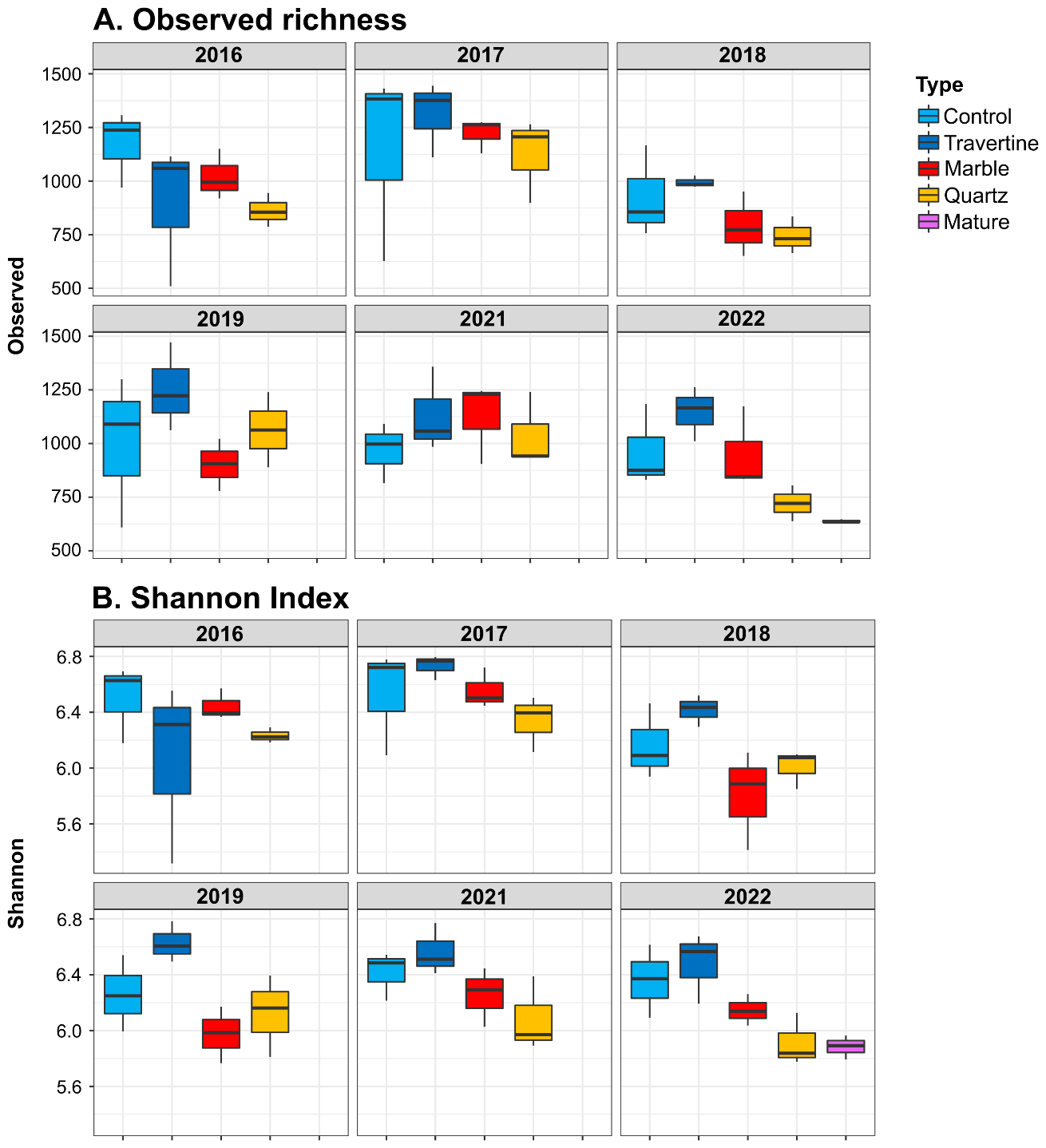


**Figure S4. Observed richness (A) and Shannon biodiversity (B) across types of samples and years for the inland array.**


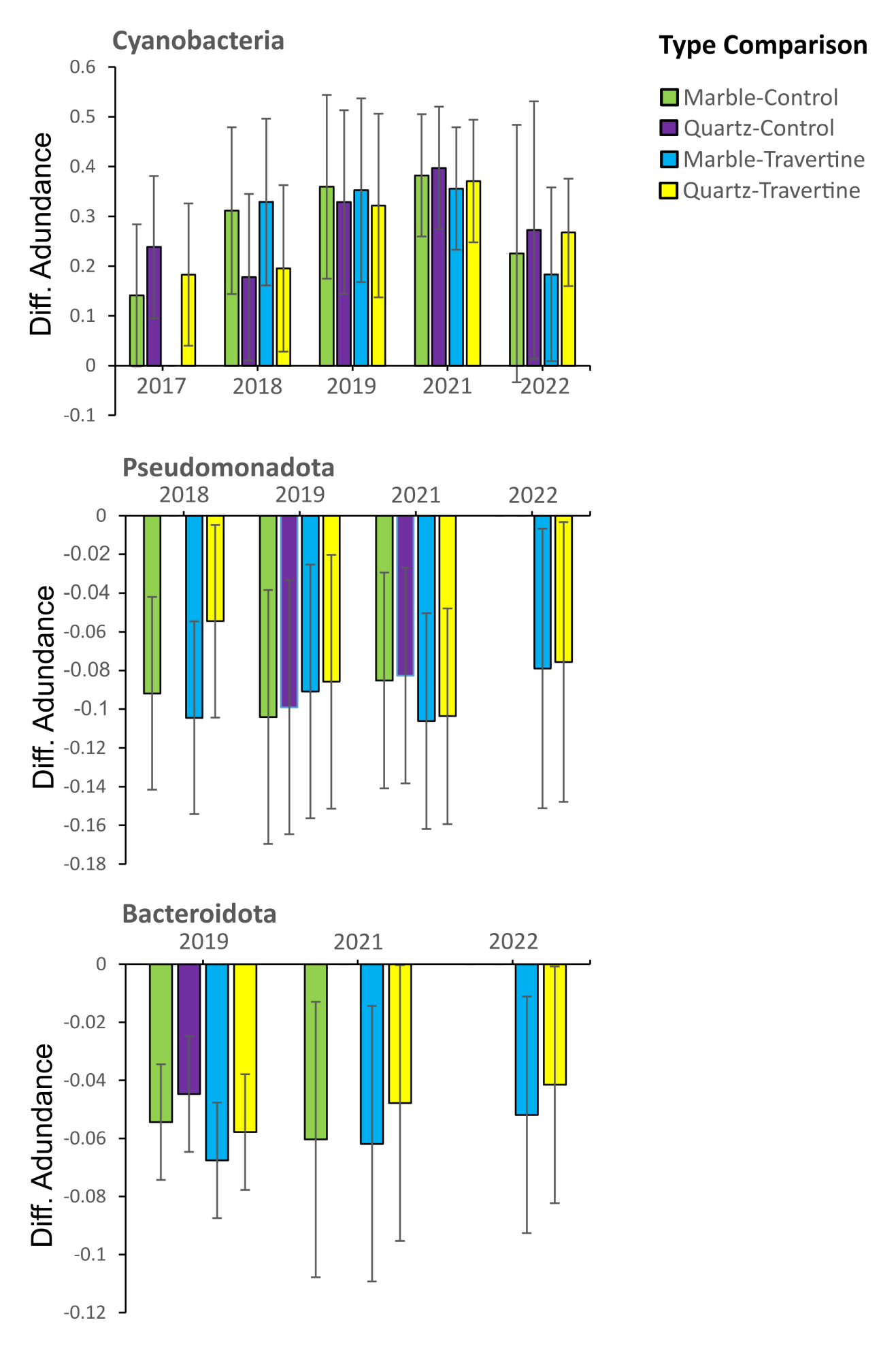


**Figure S5. Significant difference in abundances of top phyla between different groups of samples in the inland array dataset.** The significant differences were calculated using the ANOVA pair-wise comparison. Values are expressed as differences in relative abundance between the pairs, in a fraction scale between 0 and 1. Positive difference values represent higher abundances in Marble and Quartz samples, while negative values represent either abundances in Travertine and the Soil Control samples. Only significant differences (p-value < 0.05) are represented in the plots.


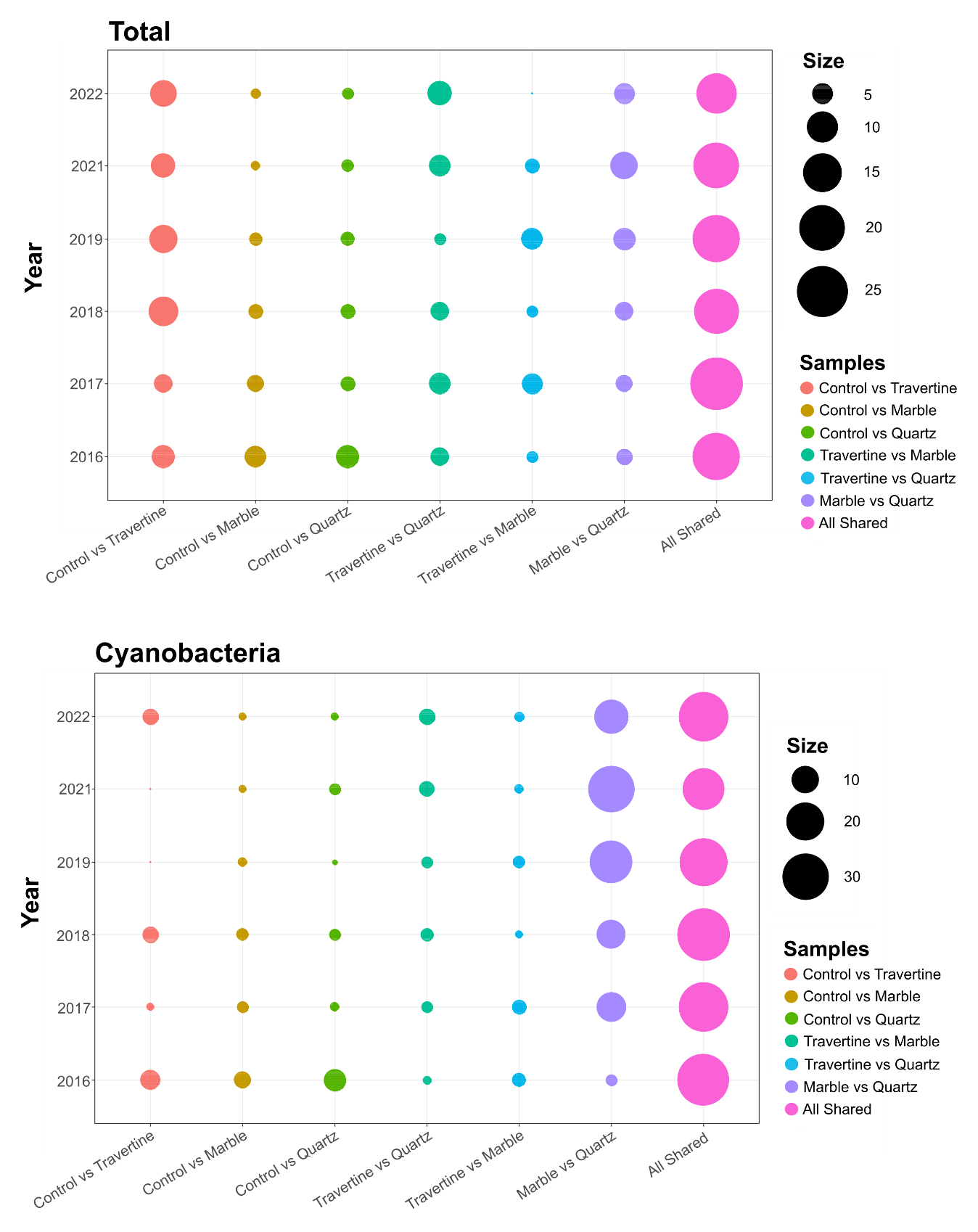


**Figure S6. Shared ASVs between sample types across the inland array.** Both the number of total shared ASVs and shared number of cyanobacterial ASVs are expressed as the percentage of the total ASV pools for both groups of ASVs. The size of the bubbles corresponds to the percentage of shared ASVs.


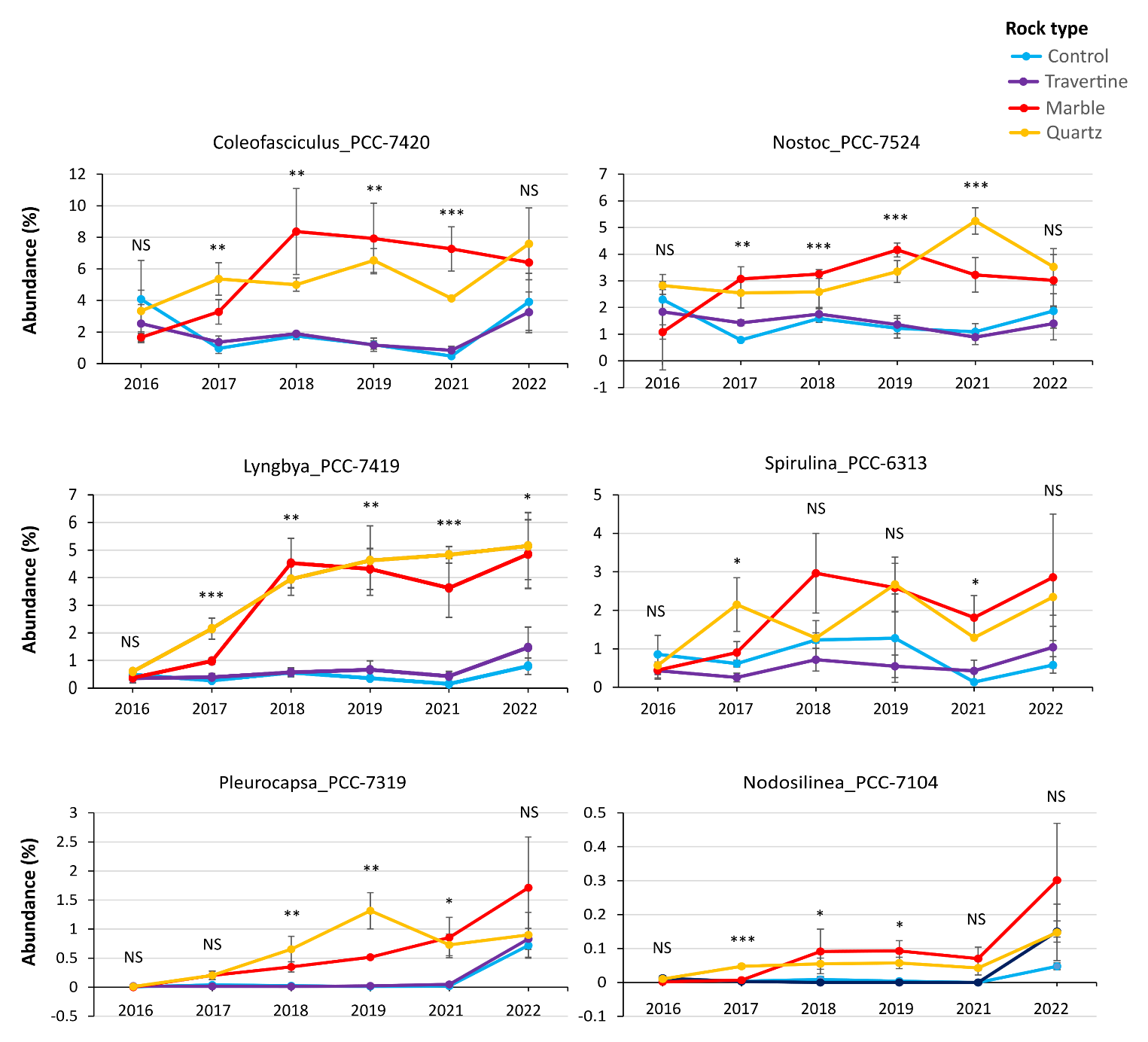


**Figure S7: Relative abundance of cyanobacterial lineages in Quartz and Marble samples across different years.** The plots represent the growth dynamics of the top six cyanobacterial genera which have been identified as over-represented in the Quartz and Marble samples at the inland array. The relative abundance (%) of these taxa was used as a proxy for the growth as a function of sampling year. Error margins for each time point were calculated as the standard deviation divided by the square root of the number of samples. Significance of differences in relative abundances between rock types at each year were calculated with anova test. Significance was highlighted using the following nomenclature: **NS** – non-significant; ***** - *p-value* < 0.05; ****** - *p-value* < 0.001; ******* - *p-value* < 0.0001.


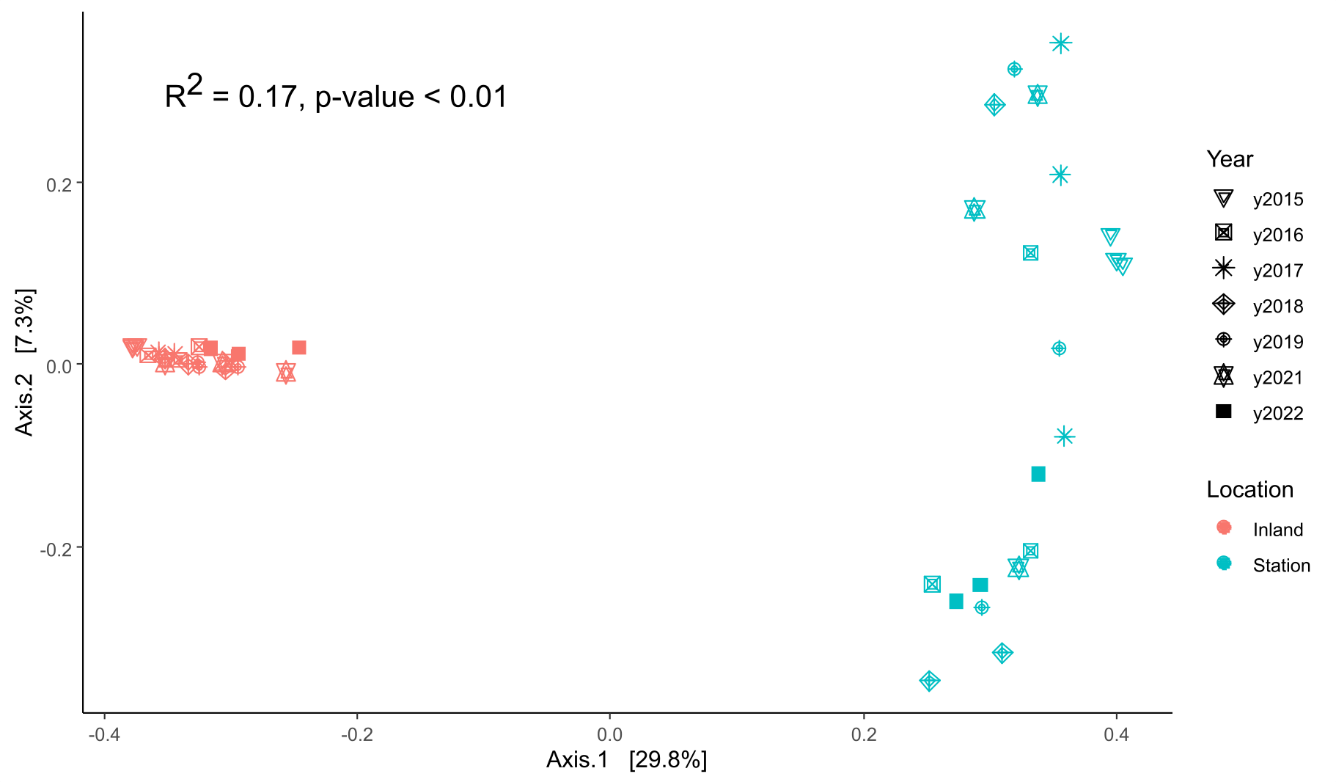


**Figure S8: Difference in microbial community structure of Soil Control samples between inland and station arrays.** The PCoA plot shows the distribution of samples according to their beta-diversity (Jaccard distance) values. Sample points are coloured according to location and shaped according to years. Samples were found to cluster significantly according to the sampling location.


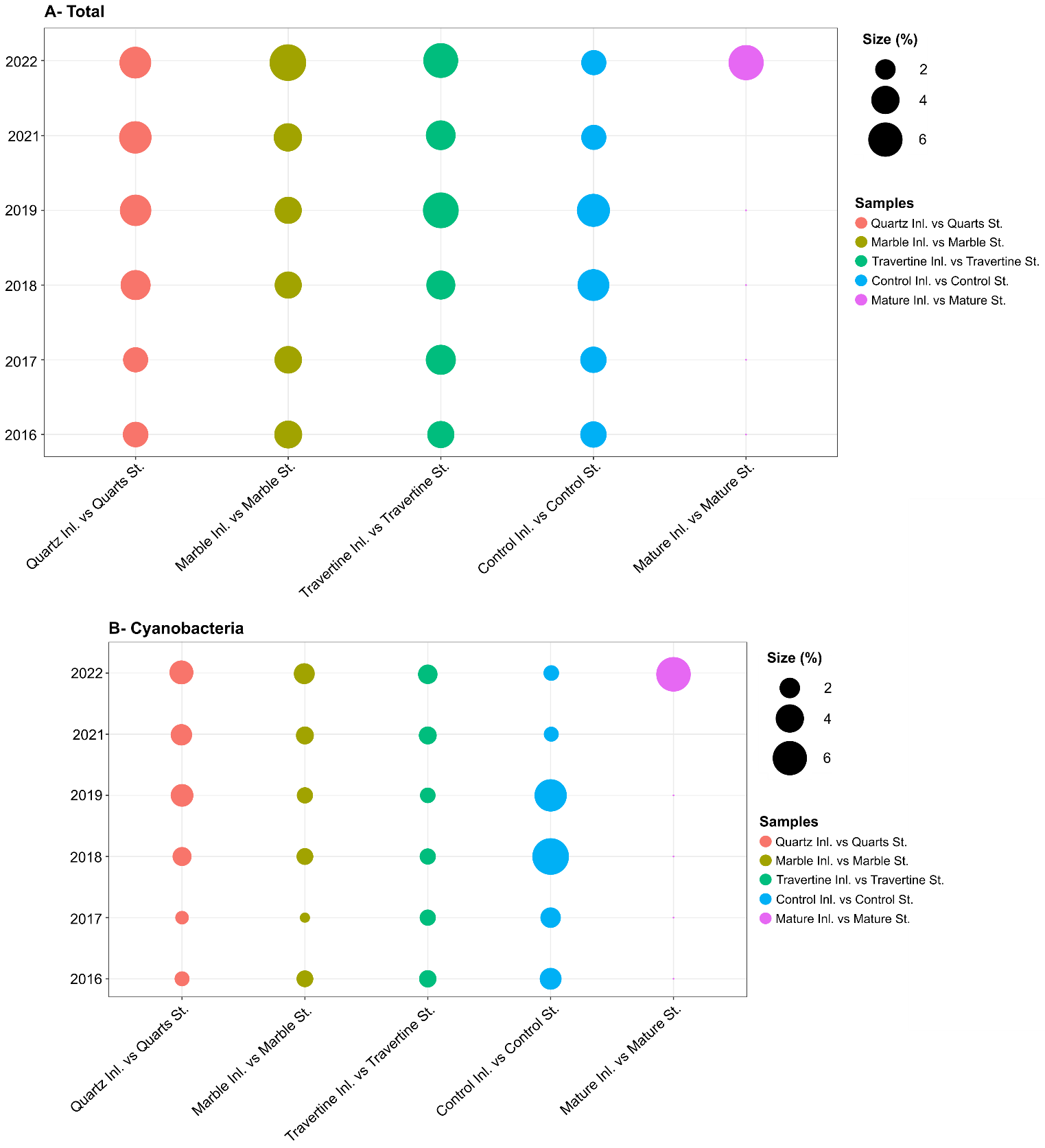


**Figure S9: Number of shared unique ASVs (A) and cyanobacterial ASVs (B) between station and inland arrays.**
